# Expansion of a core regulon by transposable elements promotes *Arabidopsis* chemical diversity and pathogen defense

**DOI:** 10.1038/s41467-019-11406-3

**Published:** 2019-08-01

**Authors:** Brenden Barco, Yoseph Kim, Nicole K. Clay

**Affiliations:** 1 Department of Molecular, Cellular and Developmental Biology, Yale University, Kline Biology Tower 734, 219 Prospect Street, New Haven, CT 06511 USA; 2 Hopkins School, 986 Forest Road, New Haven, CT 06515 USA; 3 Present Address: Seeds Research, Syngenta Crop Protection, 9 Davis Drive, Durham, NC 27703 USA

**Keywords:** Molecular evolution, DNA transposable elements, Transcriptional regulatory elements, Secondary metabolism

## Abstract

Plants synthesize numerous ecologically specialized, lineage-specific metabolites through biosynthetic gene duplication and functional specialization. However, it remains unclear how duplicated genes are wired into existing regulatory networks. We show that the duplicated gene *CYP82C2* has been recruited into the WRKY33 regulon and indole-3-carbonylnitrile (ICN) biosynthetic pathway through exaptation of a retroduplicated LINE retrotransposon (*EPCOT3*) into an enhancer. The stepwise development of a chromatin-accessible WRKY33-binding site on *EPCOT3* has potentiated the regulatory neofunctionalization of *CYP82C2* and the evolution of inducible defense metabolite 4-hydroxy-ICN in *Arabidopsis thaliana*. Although transposable elements (TEs) have long been recognized to have the potential to rewire regulatory networks, these results establish a more complete understanding of how duplicated genes and TEs contribute in concert to chemical diversity and pathogen defense.

## Abstract (web summary)

Arabidopsis plants can produce 4-hydroxyindole-3-carbonitrile (4OH-ICN) upon pathogen infection. Here, the authors show that EPCOT3, a retrotransposonderived enhancer, mediates WRKY33-binding, pathogen-responsive transcription of CYP82C2, and synthesis of 4OH-ICN.

## Introduction

Plant secondary or specialized metabolites are essential for plant survival in co-evolving biotic and fluctuating abiotic environments. The evolutionary process of chemical innovation resulted in the collective synthesis of hundreds of thousands of ecologically specialized, mostly lineage-specific metabolites^1–3^. Plant-specialized metabolic enzymes are ultimately produced from primary metabolic enzymes through gene duplication and subsequent functional divergence of one or both paralogs to produce enzymes with altered expression patterns and/or protein functions^3–5^. They are also often organized into transcription factor (TF) regulons of co-regulated genes for optimal timing, amplitude, and tissue-specific pathway gene expression and subsequent metabolite accumulation^6,7^.

Changes in *cis*-regulatory modules such as enhancers and promoters can accelerate the capture of duplicated genes into regulons, thus driving phenotypic diversity^8–10^. Enhancers consist of TF binding sites (TFBSs) and are derived either through mutation or co-option of a TFBS-carrying transposable element (TE)^10,11^. TE exaptations are hypothesized to be responsible for the rapid transcriptional rewiring of gene regulatory networks in ancient lineages of vertebrates^12–14^ and plants^15^, but general understandings of the physiological significance of this rewiring are greatly limited.

Bacteria elicit two primary immune defense modes in plants, pattern- and effector-triggered immunity (PTI and ETI)^16^. Pathogenic bacteria additionally compromise PTI via specific virulence effector proteins (effector-triggered susceptibility, ETS)^16^. PTI involves the extracellular perception of conserved molecules known as microbe-associated molecular patterns (MAMPs), whereas ETI involves the cytosolic perception of effectors. Although ETI results in the formation of more rapid and robust pathogen-specific responses including a form of programmed cell death known as the hypersensitive response (HR)^16^, both result in the ability of naive host cells to generate, through non-self perception and subsequent transcriptional reprogramming, pathogen-inducible specialized metabolites necessary for defense^17–19^.

**Fig. 1:**
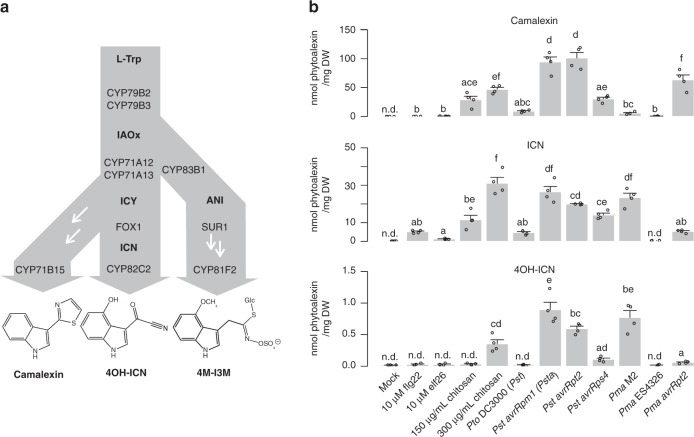
4OH-ICN is synthesized under ETI-like responses. **a** Schematic of tryptophan (L-Trp)-derived specialized metabolism in *A. thaliana*. White arrows denote the presence of additional enzymes. ANI, *aci*-nitro indole; ICY, indole cyanohydrin. **b**. LC-DAD-FLD-MS analysis of camalexin (top), ICN (middle), and 4OH-ICN (bottom) in seedlings elicited with indicated MAMPs and bacterial strains for 27 h. Data represent mean ± SE of four replicates of 15 ± 2 seedlings each. Different letters denote statistically significant differences (*P* < 0.05, one-factor ANOVA coupled to Tukey’s test). ICN totals consist of the sum of ICN and methanolic degradation product ICA-ME. 4OH-ICN totals consist of the sum of aqueous and methanolic degradation products 4OH-ICA-ME and 4OH-ICA, respectively

Three pathogen-inducible tryptophan (Trp)-derived defense metabolites— 4-methoxyindol-3-ylmethylglucosinolate (4M-I3M)^19,20^, camalexin^21,22^, and 4-hydroxyindole-3-carbonylnitrile (4OH-ICN)^23^—are known to expand innate immunity in *Arabidopsis thaliana*. Their biosynthetic pathways share an early step, which is the conversion of Trp to indole-3-acetaldoxime (IAOx) via the genetically redundant P450 monooxygenases CYP79B2 and CYP79B3^23–26^ (Fig. 1a). The camalexin and 4OH-ICN pathways additionally share the conversion of IAOx to indole-3-cyanohydrin (ICY) by partially redundant P450s CYP71A12 and CYP71A13^23,27,28^ (Fig. 1a). CYP71A13 and CYP71B15/PAD3 catalyze further reactions, leading to camalexin production^28,29^, whereas the flavin-dependent oxidase FOX1/AtBBE3 and P450 CYP82C2 convert ICY to 4OH-ICN (Fig. 1a)^23^. 4M-I3M is widely distributed across the mustard family (*Brassicaceae*), whereas camalexin is restricted to the Camelineae tribe of *Brassicaceae*^30^. The evolutionary conservation of 4OH-ICN has not yet been investigated.

The TF WRKY33 regulates the pathogen-inducible biosynthesis of camalexin in *A. thaliana*^31,32^ and its orthologs regulate numerous unrelated specialized metabolites in other flowering plant lineages^33^. The group I class of WRKYs to which WRKY33 belongs is an ancient clade of regulators; orthologs in the green alga *Chlamydomonas reinhardtii* may be ancestral to all higher plant WRKYs^33,34^. Although all WRKY TFs bind to the W-box core sequence [TTGAC(T/C)], WRKY33 preferentially binds W-boxes that are within 500 nt of the WRKY33-specific motif [(T/G)TTGAAT])^35^.

Here we show that a recent, lineage-specific TE exaptation has resulted in the expansion of a core regulon within the framework of *Arabidopsis* Trp-derived defense metabolism. Specifically, the LINE retrotransposon *EPCOT3* has retroduplicated from a WRKY33-TFBS-carrying progenitor and inserted upstream of the newly duplicated gene *CYP82C2*. Subsequent chromatin remodeling in *A. thaliana* has led *EPCOT3* to become a bona fide enhancer with demonstrated biochemical, regulatory, physiological, and fitness-promoting characteristics by way of WRKY33-binding and pathogen-responsive *CYP82C2* transcription, 4OH-ICN biosynthesis, and antibacterial defense.

## Results

### 4OH-ICN requires ETI-like responses

To identify the major Trp-derived specialized metabolites synthesized in ETI in *A. thaliana*, we compared host transcriptional and metabolic responses to the PTI-eliciting bacterial MAMPs flg22, elf26, and fungal MAMP chitosan; the PTI/ETS-eliciting pathogens *Pseudomonas syringae* pv. *tomato* DC3000 (*Pto* DC3000 or *Pst*); *P. syringae* pv. *maculicola* ES4326 (*Pma*); and the ETI-eliciting pathogens *Pst avrRpm1* (*Psta*), *Pst avrRpt2*, *Pst avrRps4*, *Pma* M2, and *Pma avrRpt2* under similar conditions as those of previous studies^19,36^. *Psm* M2 is an ETI-eliciting strain from which the *avrRpm1* gene was originally isolated^37^. Both flg22 and *Psta* induced genes involved in camalexin, 4OH-ICN, and 4M-I3M biosynthesis, with camalexin and 4OH-ICN biosynthetic genes having a higher level of induction than those of 4M-I3M in *Psta*-inoculated plants^36^ (Supplementary Table 1). On the other hand, metabolite responses between PTI and ETI differed qualitatively. 4M-I3M and its immediate precursor 4-hydroxy-I3M (4OH-I3M) were present in uninfected plants and accumulated to modest levels at the expense of parent metabolite I3M in flg22- and *Psta*-inoculated plants^19^ (Supplementary Fig. 1a). By comparison, camalexin, ICN, and 4OH-ICN were absent in uninfected plants and accumulated to high levels upon inoculation with ETI-inducing pathogens (Fig. 1b and Supplementary Fig. 1b). Furthermore, camalexin, ICN, and 4OH-ICN metabolism was greatly diminished, and 4M-I3M, 4OH-I3M, and I3M levels were mostly unchanged in the *rpm1* mutant (Supplementary Fig. 1), which is impaired in ETI recognition of *Psta*^40^. By contrast, camalexin and ICN were largely at low-to-undetectable levels in plants treated with saturating concentrations of the bacterial MAMPs flg22 and elf26^38,39^ and PTI/ETS-eliciting pathogens, with 4OH-ICN not detected in most cases (Fig. 1b). One exception was the fungal MAMP chitosan. Chitosan (150 μg/mL) induced high levels of camalexin and detectable levels of ICN (Fig. 1b), consistent with previous observations of camalexin biosynthetic gene upregulation^41^. Higher chitosan concentrations (≥ 200 μg/mL) have been shown to induce HR-like cell death in *Arabidopsis*^42^, a phenomenon commonly observed for ETI^16^. To our surprise, 300 μg/mL chitosan additionally induced detectable levels of 4OH-ICN (Fig. 1b). These results suggest that 4OH-I3M, 4M-I3M, camalexin, and ICN are synthesized in response to multiple PTI elicitors, whereas 4OH-ICN biosynthesis is specific to ETI-like responses.

### *WRKY33* is required to activate 4OH-ICN in response to *Psta*

**Fig. 2:**
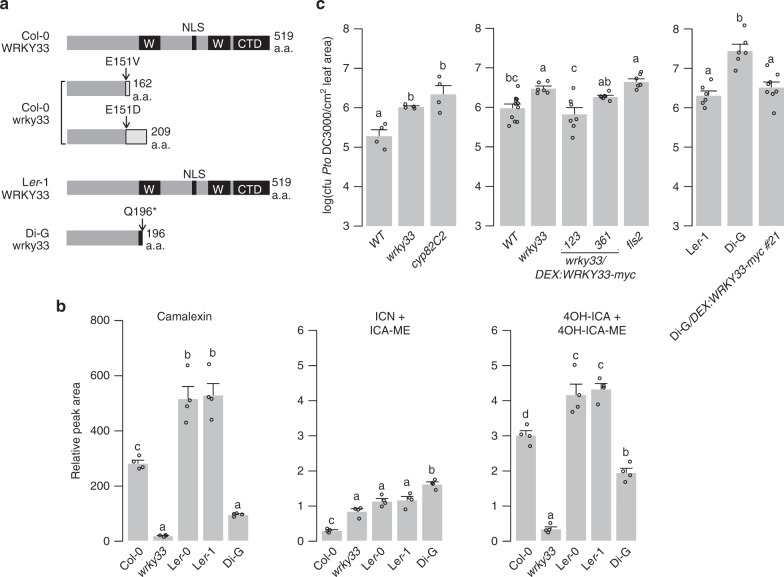
Intraspecific variation in *WRKY33* affects 4OH-ICN and immunity. **a** Schematic of WRKY33 proteins in Col-0, Col-0 *wrky33*, L*er*-1, and Di-G. Black boxes denote WRKY domains (W), nuclear localization signal (NLS), or C-terminal domain (CTD). **b** LC-DAD-MS analysis of camalexin, ICN, and 4OH-ICN in seedlings inoculated with *Psta* for 24 h. Data represent mean ± SE of four replicates of 15 ± 2 seedlings each. **c** Bacterial growth analysis of *Pst* in surface-inoculated leaves. Middle and right panels were pre-treated with 20 μM dex for 6–8 h. Data represent mean ± SE of 4 (left); 11, 6, 7, 7, 7 (middle); 6, 6, 8 (right) replicates of 15 ± 2 seedlings each. CFU, colony-forming units. Different letters in **b**, **c** denote statistically significant differences (*P* < 0.05, one-factor ANOVA coupled to Tukey’s test). Source data of Figs. 2b and 2c are provided as a Source Data file

4OH-ICN biosynthetic genes are highly co-expressed with each other^23^ and with camalexin biosynthetic genes (Supplementary Table 2), which are in the WRKY33 regulon^31,43^. To determine whether 4OH-ICN biosynthetic genes are also in the WRKY33 regulon, we compared camalexin, ICN, and 4OH-ICN levels between wild-type and a *wrky33* loss-of-function mutant that encodes two differently truncated proteins^44^ (Fig. 2a). Consistent with a previous report^31^, *wrky33* was impaired in camalexin biosynthesis in response to *Psta* and *Pst avrRps4* (Fig. 2b and Supplementary Fig. 2a). The *wrky33* mutant was similarly impaired in 4OH-ICN biosynthesis (Fig. 2b and Supplementary Fig. 2a). These results indicate that WRKY33 is required for camalexin and 4OH-ICN biosynthesis in response to multiple ETI elicitors.

To confirm that *WRKY33* is required to activate the 4OH-ICN pathway, we used a two-component glucocorticoid-inducible system to generate *wrky33* plants that in the presence of the glucocorticoid hormone dexamethasone (dex) express a wild-type copy of WRKY33 with a C-terminal fusion to 1× flag epitope (*wrky33/DEX:WRKY33-flag*; Supplementary Fig. 2b–c). Induced expression of *WRKY33-flag* restored camalexin and 4OH-ICN biosynthesis in *Psta*-challenged *wrky33* plants to greater than wild-type levels (Supplementary Fig. 2d). These results indicate that *WRKY33* is required to activate camalexin and 4OH-ICN biosynthesis in response to *Psta*.

### Natural variation in *WRKY33* affects metabolism and defense

Intraspecific variation in TFs can contribute to gain or loss of phenotypes, such as branching in maize^45^ or pelvic loss in three-spined stickleback fish^46^. In addition, the wide variation in camalexin biosynthesis reported among natural accessions of *A. thaliana*^47^ suggests that a similar variation in 4OH-ICN biosynthesis may exist. To identify additional transcriptional activators of 4OH-ICN biosynthesis that otherwise might be refractory to traditional genetic approaches, we compared intraspecific variation in *Psta*-induced camalexin, ICN, and 4OH-ICN among 35 re-sequenced accessions and *wrky33* (Col-0 accession). We found camalexin and 4OH-ICN levels to be positively correlated among accessions (*R*^2^ = 0.37; Supplementary Fig. 3a), lending further support to their co-regulation by WRKY33. Accession Dijon-G (Di-G) was identified to produce less camalexin and 4OH-ICN and more ICN than its near-isogenic relatives, the Landsberg accessions L*er*-0 and L*er*-1 (Fig. 2b and Supplementary Fig. 3a–b). In addition, differences observed in the metabolite response between Landsberg accessions and Di-G most closely resembled those between Col-0 and *wrky33* mutant (Fig. 2b and Supplementary Fig. 3a). These results led us to hypothesize that genetic variation in a regulatory gene, as opposed to an immune signaling gene, is responsible for the metabolite phenotypes observed in Di-G. To test this hypothesis, genetic variation between Di-G and three sequenced Landsberg accessions (La-0, L*er*-0, and L*er*-1) were used to identify 354 genes that were differentially mutated to high effect in Di-G (Supplementary Fig. 3c). Twenty-eight of these mutated Di-G genes were annotated by Gene Ontology to have roles in defense, including *WRKY33* (Supplementary Table 3). We confirmed by Sanger sequencing that Di-G *WRKY33* harbors a nonsense mutation early in the N-terminal DNA-binding motif (Fig. 2a), likely abolishing protein function. Our findings indicate that camalexin and 4OH-ICN are sensitive to intraspecific variation in *WRKY33*.

Camalexin and 4OH-ICN promote plant fitness by contributing non-redundantly to pathogen defense against the fitness-reducing *Pst*^23^. To confirm that disease resistance to *Pst* is also sensitive to intraspecific variation in *WRKY33*, we measured bacterial growth in adult leaves of *wrky33,* Di-G, and their respective (near-)isogenic accessions Col-0 and L*er*-1. *wrky33* and Di-G were more susceptible to *Pst* than their (near-)isogenic relatives and comparable to the 4OH-ICN biosynthetic mutant *cyp82C2*^23^ (Fig. 2c)

We additionally generated *wrky33* plants that in the presence of dex express a wild-type copy of WRKY33 with a C-terminal fusion to a larger 6× myc epitope (*wrky33/DEX:WRKY33-myc*; Supplementary Fig. 4a–c). Induced expression of WRKY33-myc in *wrky33* and Di-G plants restored and/or exceeded Col-0 and L*er*-1 levels of resistance to *Pst* (Fig. 2c) and of *Psta*-induced camalexin and ICN, respectively (Supplementary Fig. 4d–e). Together, our results support a role of *WRKY33* in pathogen defense as an activator of Trp-derived specialized metabolism.

### WRKY33 activates 4OH-ICN biosynthesis

**Fig. 3:**
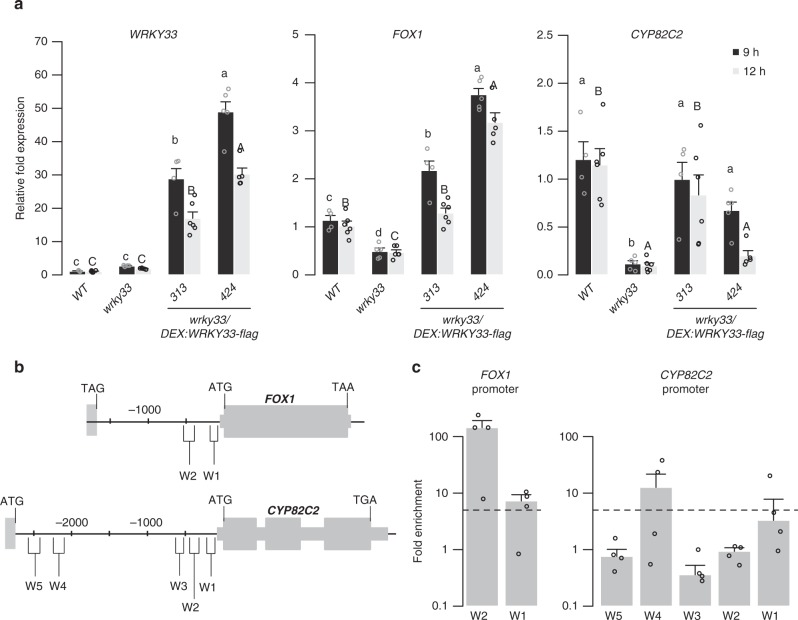
WRKY33 directly activates 4OH-ICN biosynthetic genes. **a** qPCR analysis of 4OH-ICN regulatory and biosynthetic genes in seedlings co-treated with 20 μM dex and *Psta* for 9 and 12 h. Different letters denote statistically significant differences (*P* < 0.05, one-factor ANOVA coupled to Tukey’s test). Lowercase and uppercase letters denote comparisons across 9 and 12 h time points, respectively. Data represent mean ± SE of 4, 5, 4, 5 (9 h) and 6, 6, 6, 5 (12 h) replicates of 15 ± 2 seedlings each. **b** Schematic of *FOX1* and *CYP82C2* loci, indicating nt positions of W-box-containing regions (W). **c** ChIP-PCR analysis of W-box-containing regions upstream of *FOX1* and *CYP82C2* in *wrky33/DEX:WRKY33-flag* plants co-treated with 20 μM dex (D) or mock solution (M) and *Psta* for 9 h. Dashed line represents the fivefold cutoff between weak and strong TF-DNA interactions. Data represent median ± SE of four replicates of 15 ± 2 seedlings each. Source data of Figs. 3a and 3c are provided as a Source Data file

To confirm that the 4OH-ICN biosynthetic pathway is in the WRKY33 regulon, we first compared *WRKY33*, *CYP71A13, CYP71B15, FOX1*, and *CYP82C2* transcript levels among WT, *wrky33*, *wrky33/DEX:WRKY33*-*flag*, and *wrky33/DEX:WRKY33*-*myc*. Consistent with previous reports^31^, *CYP71A13*, *CYP71B15*, and *FOX1* expression was downregulated in *wrky33* plants in response to *Psta* and upregulated in both *wrky33/DEX:WRKY33*-*flag* and *wrky33/DEX:WRKY33*-*myc* (Fig. 3a) (Supplementary Figs. 4f and 5a). Interestingly, *CYP82C2* expression and 4OH-ICN production were restored in *wrky33/DEX:WRKY33*-*flag* but not *wrky33/DEX:WRKY33-myc* or Di-G*/DEX:WRKY33-myc* plants (Figs. 2d and 3a, and Supplementary Fig. 4d–f), likely due to the interference of the larger myc tag with the WRKY33 C-terminus, a region previously linked with transactivation activity^48^. These transcriptional and metabolic findings indicate that WRKY33 mediates camalexin and 4OH-ICN biosynthesis in response to pathogen effectors.

We then tested for WRKY33-binding to W-box-containing regions upstream of camalexin and 4OH-ICN biosynthetic genes in dex-treated and *Psta*-infected *wrky33/DEX:WRKY33-flag* seedlings by chromatin immunoprecipitation (ChIP)-PCR. WRKY33 reportedly binds upstream of 4OH-ICN biosynthetic gene *CYP71A12* to a W-box region that also contains three WRKY33-specific motifs^49^. We additionally observed that *Psta*-induced WRKY33 bound strongly (greater than fivefold enrichment) upstream of 4OH-ICN biosynthetic genes *FOX1* and *CYP82C2* to W-box regions that also contain one to three WRKY33-specific motifs (W2 and W4, respectively; Fig. 3b, c and Supplementary Fig. 5b). Together with our expression analysis, our findings indicate that WRKY33 uses preferred WRKY33-binding sites to directly activate 4OH-ICN biosynthetic genes in response to pathogen effectors.

Interestingly, *Psta*-induced WRKY33 did not bind to the W5 region upstream of *CYP82C2* (Fig. 3b, c), a W-box region that does not contain any WRKY33-specific motifs and is just upstream of neighboring gene of unknown function *At4g31960*. WRKY33 reportedly binds to W5 in response to flg22^49^ and *Botrytis cinerea*^35^. By contrast, *Psta*-induced WRKY33 bound strongly to the W1 region upstream of *CYP71B15* (Supplementary Fig. 5c–d), a W-box region that also does not contain any WRKY33-specific motifs. WRKY33 reportedly binds to a region encompassing W1 in response to flg22^31,49^ and *Psta*^31^. These findings suggest that WRKY33 may use W-box extended motifs or alternative specificity motifs to target camalexin biosynthetic genes in response to pathogen effectors, or 4OH-ICN biosynthetic genes in response to MAMPs or fungal pathogens.

### *CYP82C2* underwent regulatory neofunctionalization

**Fig. 4:**
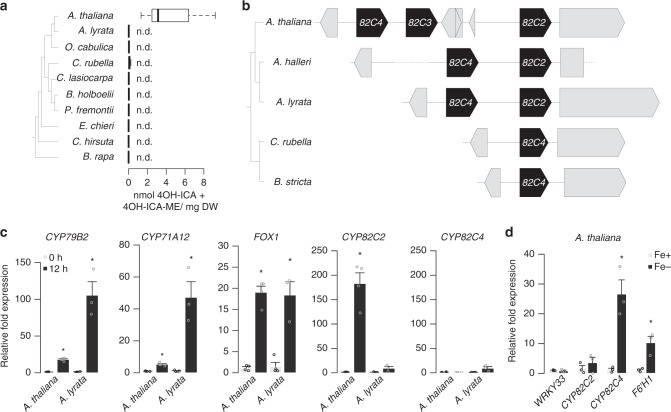
Regulatory neofunctionalization of *CYP82C2*. **a** (Left) Phylogenetic species tree. (Right) HPLC-DAD analysis of 4OH-ICN in seedlings inoculated with *Psta* for 30 h. Data in box plots represent median (center line), 25th percentile (lower box limit), 75th percentile (upper box limit), and full range of variation (whiskers) for *n* = 13, 9, 3, 6, 6, 6, 6, 3, 3, 6 replicates of 15 ± 2 seedlings each. Data were pooled from several independent experiments with *A. thaliana* as the positive control. 4OH-ICA and 4OH-ICA-ME are aqueous and methanolic degradation products of 4OH-ICN, respectively. DW, dry weight; n.d., not detected. **b** (Left) Phylogenetic species tree. (Right) Synteny map of *CYP82C* genes. Gray arrows or rectangles represent non-*CYP82C* genes. Gray dotted lines represent large ( > 500 nt) sequence gaps. **c**, **d** qPCR analysis of 4OH-ICN and sideretin biosynthetic genes in seedlings inoculated with *Psta* (c) or grown in iron-deficient medium (d). Data in **c** represent mean ± SE of 3 (12 h *Arabidopsis lyrata)* or 4 (all other) replicates of 15 ± 2 seedlings each. Data in **d** represent mean ± SE of 3 replicates of 15 ± 2 seedlings each. Asterisks denote statistically significant differences of stress-treated relative to untreated samples (*P* *<* 0.05, two-tailed *t*-test). Source data of Figs. 4a, 4c, and 4d are provided as a Source Data file

CYP82C2 catalyzes the last step in 4OH-ICN biosynthesis, hydroxylating ICN to form 4OH-ICN^23^, and likely was the last 4OH-ICN pathway gene to be recruited to the WRKY33 regulon in *A. thaliana*. To explore the phylogenetic distribution pattern of 4OH-ICN biosynthesis, we profiled ICN and 4OH-ICN metabolites in close and distant relatives of *A. thaliana* in response to *Psta*. Although ICN biosynthesis was observed across multiple close relatives, 4OH-ICN was only detected in *A. thaliana* (Fig. 4a and Supplementary Fig. 6a). This result suggests that 4OH-ICN manifests a species-specific diversification of pathogen-inducible Trp-derived metabolism in the mustard family.

In *A. thaliana*, *CYP82C2* resides in a near-tandem cluster with paralogs *CYP82C3* and *CYP82C4* (Fig. 4b). We performed phylogenetic and syntenic analyses to identify putative *CYP82C2* orthologs in a clade inclusive of ICN-synthesizing species. All identified homologs are syntenic to *CYP82C2* or *CYP82C4*, and encode proteins with > 88% identity to one another (Fig. 4b and Supplementary Fig. 6b–c). *CYP82C3* is present only in *A. thaliana* and, although more similar to *CYP82C2* than *CYP82C4* in sequence (Fig. 4b and Supplementary Fig. 6b), it is not functionally redundant with *CYP82C2*^23^. *CYP82C4* is required for the biosynthesis of sideretin, a widely conserved, phenylalanine-derived metabolite required for iron acquisition^50^. *CYP82C4* has syntenic orthologs in the mustard family (Fig. 4b and Supplementary Fig. 6b), correlating with the distribution of sideretin biosynthesis^50^. By contrast, *CYP82C2* has syntenic orthologs only within the *Arabidopsis* genus (Fig. 4b and Supplementary Fig. 6b). These results suggest that *CYP82C2* duplicated from *CYP82C4* prior to the formation of the *Arabidopsis* genus and then acquired a new expression pattern and/or catalytic function prior to *A. thaliana* speciation ~2 million years later^51,52^.

CYP82C2 and CYP82C4 were previously characterized to 5-hydroxylate with equal efficiency the specialized metabolite 8-methoxypsoralen, a molecule structurally reminiscent of ICN and sideretin^53^. The apparent similarities in substrate specificity and catalytic function suggest that *CYP82C2* may have diverged from *CYP82C4* in expression but not protein function. To test this, we first compared the expression of *CYP82C2* and *CYP82C4* in *A. lyrata* and *A. thaliana* in response to *Psta*. 4OH-ICN biosynthetic genes *CYP79B2, CYP71A12*, and *FOX1* were upregulated in both species, consistent with the common presence of ICN (Fig. 4a, c). By contrast, *CYP82C2* levels were respectively upregulated and unchanged in *A. thaliana* and *A. lyrata*, correlating with the distribution of 4OH-ICN in these species (Fig. 4a, c). *CYP82C4* expression was unchanged in both species (Fig. 4c). These results indicate that 4OH-ICN biosynthesis is linked with pathogen-induced expression of *CYP82C2*.

We then compared the aligned upstream sequences of *CYP82C2* and *CYP82C4* in *A. lyrata* and *A. thaliana*, and observed good sequence conservation among orthologs but poor conservation among paralogs (Supplementary Fig. 6d), indicating that sequences upstream of *CYP82C4* and *CYP82C2* were independently derived. We performed expression analysis in *A. thaliana* to confirm that *CYP82C2* and *CYP82C4* have different expression patterns. Consistent with previous reports^23,50^, *CYP82C2* expression is upregulated in response to *Psta* and unchanged under iron deficiency, whereas *CYP82C4* is upregulated under iron deficiency and unchanged in response to *Psta* (Fig. 4c, d and Supplementary Table 1). Finally, *CYP82C4* expression is unchanged in *Psta*-challenged, dex-induced *wrky33* and *wrky33/DEX:WRKY33-flag* (Supplementary Fig. 6e). Our findings suggest that *CYP82C2* diverged from *CYP82C4* by acquiring WRKY33 regulation for its pathogen-induced expression.

We next assessed dN/dS ratios along branches of the CYP82C phylogenetic tree (Supplementary Fig. 6b) and found good support for purifying selection acting on CYP82C enzymes (*ω* = 0.21) and no support for positive selection acting on CYP82C2/3 enzymes (Supplementary Data 1). Lastly, we identified non-conserved amino acid residues among CYP82C homologs and mapped this information onto a homology model of CYP82C2. The protein inner core, which encompasses the active site and substrate channel, is highly conserved among CYP82C homologs (Supplementary Fig. 6f), and is consistent with CYP82C2 and CYP82C4’s reportedly redundant catalytic functions^53^. Altogether, our findings suggest that *CYP82C2* underwent regulatory neofunctionalization, diverging from *CYP82C4* in expression but not protein function.

### TE *EPCOT3* is a *CYP82C2* enhancer

**Fig. 5:**
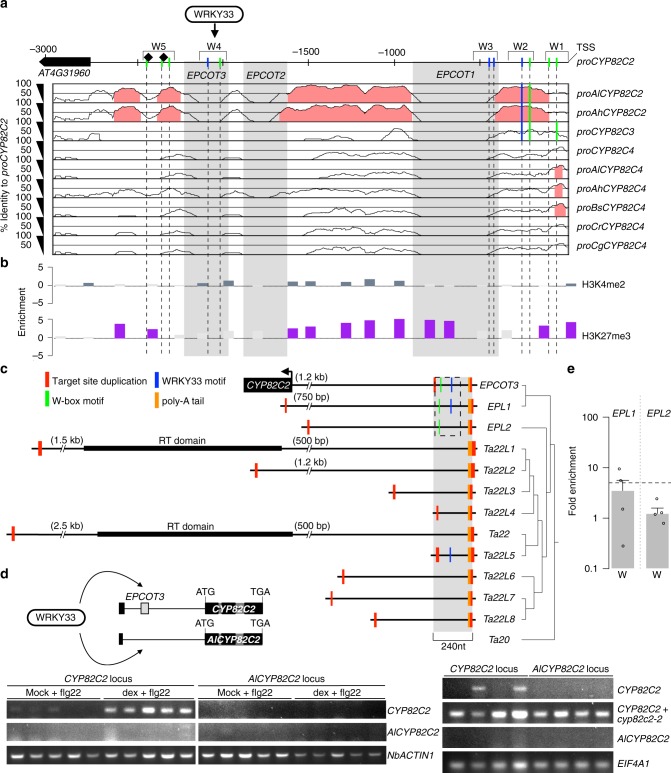
TE *EPCOT3* is a *CYP82C2* enhancer. **a** mVISTA plot of *CYP82C2* upstream sequence, indicating nt positions of unique (*EPCOT1–3;* gray boxes) and conserved regions (≥ 70% identity, pink) among homologous sequences. Also indicated are positions of W-boxes (green) and WRKY33-specific motifs (blue) present (solid lines) or absent (dashed lines) in each homologous sequence, known WRKY33 TFBSs (diamonds) and ChIP-tested regions (W1–5). *Al, A. lyrata*; *Ah*, *Arabidopsis halleri*; *Bs*, *Boechera stricta*; *Cg*, *Capsella grandiflora*; *Cr*, *Capsella rubella*; TSS, transcriptional start site. **b** Epigenetic map of *CYP82C2* upstream sequence, indicating positions of significant H3K4me2 (blue–gray bars) and H3K27me3 (purple bars). **c** (Left) Schematic of *EPCOT3* and related LINE retrotransposons in *A. thaliana*, indicating positions of *CYP82C2* and reverse-transcriptase (RT) domains. See also Supplementary Note 1. Dashed box outlines W-boxes (green lines) and/or WRKY33-binding motifs (blue lines) within *EPCOT3*/*EPL*s. (Right) Phylogenetic maximum likelihood tree. **d** (Upper left) Schematic of *CYP82C2* and *AlCYP82C2* transgenic loci used for WRKY33 transactivation experiments. (Lower left) RT-PCR images of *CYP82C2*, *AlCYP82C2*, and *NbACTIN1* in *N. benthamiana* leaves co-transfected with *DEX:WRKY33-flag* and *CYP82C2* or *AlCYP82C2* locus, and incubated with 1 μM flg22 and mock solution (0.5% DMSO) or 20 μM dex for 30 h (*CYP82C2*/*AlCYP82C2*) or 24 hr (*NbACTIN1*). Data represent five replicates (three leaf discs each). (Lower right) RT-PCR images of *CYP82C2*, *AlCYP82C2*, and *EIF4A1* in *A. thaliana cyp82C2* protoplasts transfected with *CYP82C2* or *AlCYP82C2* locus and elicited 6 h with 1 μM flg22. As original *CYP82C2* primers detect endogenous transcription downstream of the *cyp82C2* T-DNA insertion (see *CYP82C2* + *cyp82C2-2*, second row), a second set of primers (CYP82C2*, Supplementary Data 2) flanking the insertion was used to test WRKY33 transactivation (see *CYP82C2*, first row). Data represent four replicates of 2.5 × 10^5^ protoplasts each. **e** ChIP-PCR analysis of W-box-containing regions (W) within *EPL*s in *wrky33/DEX:WRKY33-flag* plants co-treated 9 h with 20 μM dex or mock solution and *Psta*. Data represent median ± SE of four replicates (~210 seedlings each). Dashed line represents fivefold cutoff between weak and strong TF-DNA interactions. Source data of Figs. 5d and 5e are provided as a Source Data file

WRKY33 regulation of *CYP82C2* is mediated by a WRKY33-TFBS in the W4 region (Figs. 3 and 5a; Supplementary Fig. 5c). Preferential WRKY33-binding at this region should also be influenced by chromatin features associated with *cis*-regulatory elements such as enhancers and basal promoters^54^. To investigate how *CYP82C2* acquired WRKY33-binding for its pathogen-induced expression, we compared the aligned upstream sequences of *CYP82C* homologs within a clade inclusive of ICN-synthesizing species. We observed three large upstream sequences specific to *A. thaliana CYP82C2*, hereafter named *Eighty-two-C2 Promoter Contained Only in A. Thaliana1-3* (*EPCOT1–3*; Fig. 5a). *EPCOT3* in particular is a 240 nt region that completely encompasses W4 (Fig. 5a), indicating that WRKY33’s regulation of *CYP82C2* in response to *Psta* may be species-specific. Further bioinformatics analysis revealed that *EPCOT3* is enriched with the activating histone mark H3K4me2 and lacks the repressive histone mark H3K27me3 (Fig. 5b)^55,56^, which are epigenetic signatures of an active enhancer^57–59^. Our findings suggest that *EPCOT3* functions as an enhancer that mediates WRKY33-binding and activation of *CYP82C2* in response to pathogen effectors.

*EPCOT3* contains a 3′-poly-A tail and is flanked by variable-length target site duplications (Fig. 5c and Supplementary Fig. 7a), which are hallmarks of eukaryotic LINE retrotransposons^60^. LINE retrotransposition (reverse transcription and integration) results in frequent 5′-truncation of retrocopies^61^. We identified 11 variably truncated retrocopies similar to *EPCOT3* throughout the genome (Fig. 5c, Supplementary Fig. 7a–b, and Supplementary Table 4), including *Ta22*, one of the first LINEs characterized in *A. thaliana*^62^. *EPCOT3*-related LINEs were sorted into two groups roughly correspondent to their phylogenetic placement: *EPCOT3-LIKE* (*EPL*) for those with high identity (> 65%) to *EPCOT3*, and *Ta22* or *Ta22*-*LIKE* (*Ta22L*) for the remainder (Supplementary Fig. 7a and Supplementary Table 4). Only *Ta22* and *Ta22L1* are full-length LINEs (Fig. 5c), presumably encoding the proteins necessary for their own transposition and for the transposition of non-autonomous family members such as *EPCOT3*. Through synteny analysis, we also identified two species-specific *Ta22L*s, but no *EPLs*, in *A. lyrata* (Supplementary Table 4). To confirm the involvement of *EPCOT3* in species-specific expression of *CYP82C2*, we introduced WRKY33 into *Nicotiana benthamiana* (tobacco) leaves and *A. thaliana cyp82C2* protoplasts transfected with either the *A. thaliana* or *A. lyrata CYP82C2* locus (coding and 3000 nt upstream sequences, Fig. 5d). We observed transactivation by WRKY33 of the *A. thaliana* gene, but not that of *A. lyrata* (Fig. 5d and Supplementary Fig. 7d). Altogether, these data indicate that *EPCOT3* and *EPLs* arose from retrotransposition following the speciation of *A. thaliana*, and that the *EPCOT3*-containing *A. thaliana CYP82C2* promoter is sufficient to confer WRKY33-mediated transcription of *CYP82C2*.

Of the *EPL* retrocopies, *EPL1* is most similar to *EPCOT3* (85.4% identity), sharing the W-box and WRKY33-specific motif, whereas *EPL2* is less similar (67%) and lacks the WRKY33-specific motif (Fig. 5c, Supplementary Fig. 7a, and Supplementary Table 4). *EPL1* and *EPL2* are much less truncated than *EPCOT3* (Fig. 5c) and lack epigenetic signatures typical of *cis*-regulatory sequences^55,56^ (Supplementary Fig. 7c). To investigate whether the sequences and chromatin features associated with *EPL*s are sufficient for WRKY33 binding, we tested for WRKY33 binding to *EPL* sequences homologous to the W4 region of *EPCOT3* in dex-treated, *Psta*-infected *wrky33/DEX:WRKY33-flag* plants by ChIP-(q)PCR. Compared with *EPCOT3* (Fig. 3c), WRKY33 bound weakly or not at all to *EPL1* and *EPL2*, respectively (Fig. 5e, and Supplementary Fig. 7e). Our findings suggest the following history: (1) *EPL1* likely retroduplicated from *EPL2* or its progenitor, which already contained a W-box; (2) *EPL1* then acquired a WRKY33-specific motif by mutation; and (3) *EPCOT3* retroduplicated from *EPL1* and then acquired epigenetic signatures of an enhancer, thereby allowing selection to act on standing variation rather than de novo mutation for *CYP82C2* recruitment into the 4OH-ICN biosynthetic pathway.

## Discussion

**Fig. 6:**
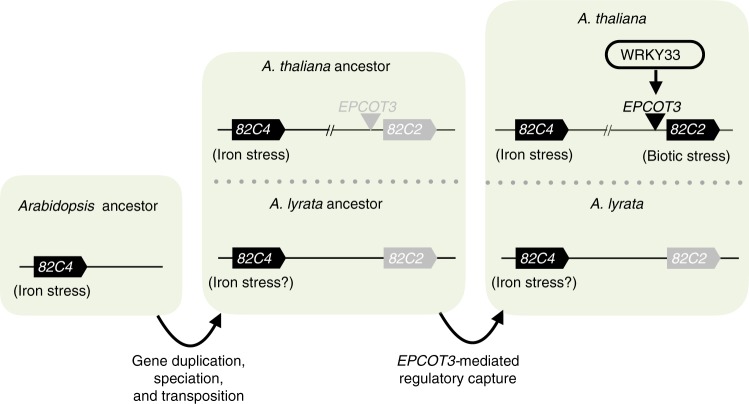
Model of regulatory neofunctionalization of *CYP82C2*. An ancestral gene with roles in iron-stress responses (*CYP82C4*) underwent gene duplication in a progenitor species to *A. thaliana* and *A. lyrata*, leading to ancestral *CYP82C2*. Subsequent speciation led to ancestral *A. thaliana* and *A. lyrata*. In the former species, a significant degree of retroduplication, mutagenesis, and transposition events occurred, culminating with the formation of W-box and WRKY33-specific sequences in the ancestral *EPCOT3* and its integration upstream of *CYP82C2*. Subsequent epigenetic modifications in *A. thaliana* were necessary to permit WRKY33 binding and *CYP82C2* activation. Features in black have a hypothesized function, whereas features in gray have no known function. Double-dashed line indicates features omitted from view (e.g., *CYP82C3*)

TEs were originally conceived to act as controlling elements of several loci in the genome^63^, and exaptation of TEs into *cis*-regulatory modules has been hypothesized to be responsible for the rapid transcriptional rewiring in more ancient lineages of vertebrates^12–14^. However, few (if any) evolutionarily recent TE exaptation events in vertebrates and higher plants have been demonstrated to have biochemical, regulatory, physiological, and fitness-promoting functions^14^. With well over a dozen genomes available including the genetic model *A. thaliana*, the mustard family presents an excellent system for examining such events. In this study, we show that *EPCOT3* is a TE-derived enhancer that mediates WRKY33 binding, pathogen-responsive transcription of *CYP82C2*, synthesis of the species-specific metabolite 4OH-ICN, and pathogen defense (Fig. 6). These results demonstrate how a recent TE exaptation can wire a new gene into an ancient regulon, ultimately leading to a positive effect on fitness.

Although the *EPL1/EPCOT3* progenitor retrotransposed a preferred WRKY33-TFBS in the form of *EPCOT3* upstream of *CYP82C2*, a further series of epigenetic modifications were needed to facilitate optimal access of *EPCOT3* by WRKY33 (Fig. 6). *EPL1* exists in a silenced heterochromatin state^55,56^ (Supplementary Fig. 7c), typical for TEs^64^, and is bound weakly by WRKY33 (Fig. 5e), whereas *EPCOT3* is in an open chromatin state^55,56^ (Fig. 5b) and bound relatively strongly by WRKY33 (Fig. 3c). The more severe 5′-truncation of *EPCOT3* could account for its release from TE-silencing mechanisms and the initially weak WRKY33 binding could provide a seed for chromatin remodelers to drive the exaptation of newly retrotransposed *EPCOT3* into a bona fide enhancer. Further epigenomic sampling within *Arabidopsis* is needed to better clarify the epigenetic transformations underlying the *EPCOT3* exaptation event.

Compared with closely related Landsberg accessions (Supplementary Fig. 3), Di-G synthesizes less camalexin and 4OH-ICN^47^ (Fig. 2b), and is more susceptible to a range of bacterial and fungal pathogens^47,65^ (Fig. 2c). WRKY33 has been implicated in camalexin biosynthesis^31^ and antifungal defense^44^. We identified WRKY33 as causal for some if not all of these phenotypes in Di-G. In addition, WRKY33’s involvement in antibacterial defense is consistent with the contribution of camalexin and 4OH-ICN toward antibacterial defense^23^.

WRKY33 is an ancient TF responsible for many fitness-promoting traits in plants; thus, it is unexpected that an *A. thaliana* accession would have a naturally occurring *wrky33* mutation (C536T transversion). Di-G is the sole member of 1,135 sequenced accessions to have a high-effect single-nucleotide polymorphism (SNP) in *WRKY33*^66^, and may have originated from a L*er*-0 ethyl methanesulfonate (EMS) mutagenesis screen, whose mutagenesis rate^67^ is well within the range of ~25,000 SNPs that are not concordant between Di-G and L*er*-0^66^ (Supplementary Fig. 2f). However, features of EMS mutations (i.e., transversion mutations) or X-ray mutations (i.e., indels) are not enriched in the Di-G pseudogenome relative to related pseudogenomes (Supplementary Table 5). These findings suggest that the *wrky33* Di-G mutation is naturally derived.

## Methods

### Plant materials and growth

For quantitative PCR (qPCR) and high-performance liquid chromatography coupled with diode array detection and fluorescence detection (HPLC-DAD-FLD) analyses, surface-sterilized *A. thaliana* accession Columbia-0 (Col-0) seeds were sown in 12-well microtiter plates sealed with Micropore tape (3 M, St. Paul, MN), each well containing ~15 ± 2 seeds and 1 mL of either filter-sterilized 1× Murashige and  Skoog media (pH 5.7–5.8) (4.43 g/L Murashige and Skoog basal medium with vitamins [Phytotechnology Laboratories, Shawnee Missions, KS], 0.05% MES hydrate, 0.5% sucrose) or iron-deficient media (amounts per liter): sucrose, 5.0 g; potassium nitrate, 1.9 g; ammonium nitrate, 1.65 g; MES monohydrate, 0.5 g; calcium chloride dihydrate, 0.44 g; magnesium sulfate heptahydrate, 0.37 g; monopotassium phosphate, 0.17 g; myo-inositol, 0.1 g; disodium EDTA, 29.2 mg; manganese sulfate monohydrate, 16.9 mg; zinc sulfate heptahydrate, 8.6 mg; boric acid, 6.2 mg; glycine, 2.0 mg; potassium iodide, 0.83 mg; nicotinic acid, 0.5 mg; pyridoxine hydrochloride, 0.5 mg; sodium molybdate dihydrate, 0.25 mg; thiamine hydrochloride, 0.1 mg; cobalt chloride hexahydrate, 25.0 μg; and copper sulfate pentahydrate, 25.0 μg. On day 9, seedlings were transferred to 6-well microtiter plates, each well containing ~15 seeds and 3 mL Murashige and Skoog or iron-deficient media. For *Polyctenium fremontii*, surface-sterilized seeds were sown on Murashige and Skoog agar plates. For all other species, surface-sterilized seeds were sown in 6-well plates, each well containing ~15 seeds and 3 mL Murashige and Skoog media. On day 9, media were refreshed prior to bacterial elicitation. Microtiter plates were placed on grid-like shelves over water-filled trays on a Floralight cart (Toronto, Canada) and plants were grown at 21 °C with 60% humidity under a 16 h light cycle (70–80 μE m^−2^ s^−1^ light intensity). For ChIP analyses, ~200 surface-sterilized seeds were sown in a 100 × 15 mm petri plate containing 20 mL of 1× Murashige and Skoog media. Media were exchanged for fresh media on day 9. For bacterial infection assays, plants were grown on soil (3:1 mix of Farfard Growing Mix 2 [Sun Gro Horticulture, Vancouver, Canada] to vermiculite [Scotts, Marysville, OH]) at 22 °C daytime/18 °C nighttime with 60% humidity under a 12 h light cycle [50 (dawn/dusk) and 100 (midday) μE m^−2^ s^−1^ light intensity]. Seed stock information is shown in Supplementary Table 6.

### Vector construction and transformation

To generate the *DEX:WRKY33-flag* construct, *WRKY33* was PCR-amplified from genomic DNA using the primers WRKY33gXhoF (5′-AACTCGAGAAGAACAAGAACCATCAC-3′) and W33flagSpeR (5′-CGACTAGTCTACTTGTCGTCATCGTCTTTGTAGTCGGGCATAAACGAATCGAAA-3′), and subcloned into the *Xho*I/*Spe*I sites of pTA7002 vector^68^. To generate the *DEX:WRKY33-myc* construct, *WRKY33* was PCR-amplified using the primers WRKY33gXhoF and WRKY33gStuR (5′-AAGGCCTGGCATAAACGAATCGAAAAATG-3′), and subcloned into the *Xho*I/*Stu*I sites of pTA7002-6x c-Myc vector^69^. Constructs were introduced into *wrky33* and Di-G plants via *Agrobacterium tumefaciens*-mediated floral dip method^70^ and transformants were selected on agar media containing 15 μg/mL hygromycin B (Invitrogen, Carlsbad, CA).

To generate the *CYP82C2* locus construct, the *CYP82C2* upstream and coding sequences were PCR-amplified from *A. thaliana* genomic DNA using the primers At82C2proXbaF (5′-GCTCTAGAAGCTTCCAATAAAACATTC-3′) and At82C2proBamR (5′-GCGGATCCAGTGGTTTGAGCGTGCAAA-3′), and At82C2geneBamF (5′-GCGGATCCATGGATACTTCCCTCTTTTC-3′) and At82C2geneSmaR (5′-TTCCCGGGCTACTTGTCGTCATCGTCTTTGTAGTCCACATAAAGCCCTTCCTTAAG-3′). Sequences were subcloned into the *Xba*I*/Sma*I sites of pBI101 vector^71^. To generate the *AlCYP82C2* locus construct, the *AlCYP82C2* upstream and coding sequences were PCR-amplified from *A. lyrata* genomic DNA using the primers Al82C2proSalF (5′-CGGTCGACTATTCCAGGAGCATACAA-3′) and Al82C2proBglIIR (5′-GGAGATCTAATGTTTTAAAAGTGCAAAAGAG-3′), and Al82C2geneBamHF (5′-GCGGATCCATGGATACATCCCTCTTTTC-3′) and Al82C2geneSmaR (5′-TTCCCGGGCTACTTGTCGTCATCGTCTTTGTAGTCCACAAAAAGTTCTTCCTTAAGAC-3′), and subcloned into the *Sal*I/*Sma*I sites of pBI101 vector. *DEX:WRKY33-flag*, *CYP82C2*, and *AlCYP82C2* constructs were introduced into *N. benthamiana* leaves as previously described^23^ with the following modifications: leaves were infiltrated with transformed *Agrobacterium* strains that were grown in lysogeny broth (LB) medium supplemented with 30 μg/mL gentamycin and 50 μg/mL kanamycin to an OD_600_ of 0.7. Sixteen hours post-*Agro*-infiltration, leaves were sprayed with 20 μM dex, 0.1% Tween-20, and 1 µM flg22, and incubated for 24 and 30 h. Three 8 mm leaf discs were pooled per sample and snap-frozen for reverse-transcriptase PCR (RT-PCR) analyses. *CYP82C2* and *AlCYP82C2* constructs were introduced into *A. thaliana cyp82C2* via PEG-mediated protoplast transformation^72^ with the following modifications: 2.5 × 10^5^ protoplasts were transfected with 3 μg of construct for 20 min, recovered in 2.5× volume of W5 solution, elicited with 1 μM flg22 in 1 mL W5 solution for 6 h, and snap-frozen for RT-PCR analyses.

### Bacterial infection and MAMP elicitation

A single colony of *P. syringae* pv. *maculicola* (*Pma*) M2 (containing *avrRpm1*, but not *avrRps4* or *avrRpt2*), *Pma* ES4326 (containing no aforementioned effectors), *Pma* ES4326 *avrRpt2*, *P. syringae* pv. *tomato* DC3000 (*Pst*, containing no aforementioned effectors), *Pst avrRpm1*, *Pst avrRps4*, and *Pst avrRpt2* from a freshly streaked 3-day-old agar plate was used to inoculate 2 mL of LB medium containing appropriate antibiotics. Strains were grown to log phase, washed in sterile water twice, resuspended in sterile water to OD_600_ of 0.2, and incubated at room temperature with no agitation for 3 to 6 h, prior to infection. Chitosan (90% deacetylated chitin; Spectrum Chemical Mfg Corp, New Brunswich, NJ) was prepared in 0.1 N acetic acid and neutralized with 0.1 N NaOH to pH 5.8, to a stock concentration of 1.2 mg/mL.

Hydroponically grown 9-day-old seedlings were inoculated with bacterial strains to OD_600_ of 0.013 or treated with 10 μM flg22 (QRLSTGSRINSAKDDAAGLQIA; Genscript, Nanjing, China, 10 μM elf26 (ac–SKEKFERTKPHVNVGTIGHVDHGKTT; Genscript), and 150 or 300 μg/mL chitosan. Seedlings were snap-frozen 9 h post infection for ChIP analyses, 12 h post infection for qPCR analyses, and 24–28 h post infection for HPLC-DAD analyses.

Four- to-five-week-old adult leaves were treated with 0.0125% Silwet (Phytotechnology Laboratories) or 0.0125% Silwet and 20 μM dex for 20 s, and incubated on 0.8% (w/v) tissue-culture agar plates on a light cart at 21 °C for 6–8 h. Leaves were then surface-inoculated with *Pst* (OD_600_ = 0.002 or 10^6^ colony-forming units (cfu)/cm^2^ leaf area) in the presence of 0.01% (v/v) Silwet L-77 for 15 s and incubated on 0.8% (w/v) tissue-culture agar plates at 21 °C under a 16 h light cycle (70–80 μE m^−2^ s^−1^ light intensity) for 3 days. Leaves were then surface-sterilized in 70% ethanol for 10 s, rinsed in sterile water, surface-dried on paper towels, and the bacteria were extracted into water, using an 8 mm stainless steel bead and a ball mill (20 Hz for 3 min). Serial dilutions of the extracted bacteria were plated on LB agar plates for cfu counting.

### RNA isolation and RT-PCR

Total RNA was extracted from snap-frozen seedlings using TRIzol reagent (Invitrogen) and 2.5 µg was reverse-transcribed with 3.75 µM random hexamers (Qiagen), 20 units of M-MuLV (New England Biolabs), and 20 units of ProtoScript II (New England Biolabs) for 1 h at 42 °C and then for 15 min at 70 °C. Resulting cDNA:RNA hybrids were treated with 10 units of DNase I (Roche) for 30 min at 37 °C and purified on PCR clean-up columns (Macherey–Nagel). qPCR was performed with Kapa SYBR Fast qPCR master mix (Kapa Biosystems) and CFX96 real-time PCR machine (Bio-Rad). Biological and technical replicates were performed on the same 384-well PCR plate, and *EIF4A1* (*AT3G13920*) and *AlEIF4A1* (*AL3G26100*) housekeeping genes were used to normalize mRNA levels between different samples. Primer sequences and efficiencies are listed in Supplementary Data 2. Total RNA from tobacco leaf discs and *A. thaliana* protoplasts was extracted from snap-frozen samples using 300 µL of Cell Lysis Solution (2% (w/v) SDS, 63 mM sodium citrate, 132 mM citric acid, 1 mM EDTA), 100 µL of Protein-DNA Precipitation Solution (4 M NaCl, 16 mM sodium citrate, 32 mM citric acid), and 300 µL of isopropanol. 2 μg RNA was then reverse-transcribed  and complementary DNA was diluted 7.5-fold. Four microliters of cDNA was used in 20 μL PCR reactions and resulting PCR products were separated on 2% agarose gels. PCR was performed on C1000 thermal cycler (Bio-Rad) with the following thermal cycling program: 95 °C for 3 min; 40 cycles of 95 °C for 10 s, 53 °C for 15 s, and 72 °C for 7 s (*WRKY33*, *CYP82C2*, *AlCYP82C2*), 15 s (*NbACTIN1*), or 21 s (CYP82C2*). Primer sequences are listed in Supplementary Data 2.

### Camalexin and 4OH-ICN extraction and LC-DAD-MS

Snap-frozen seedlings were lyophilized, weighed, and homogenized using a 5 mm stainless steel bead and ball mill (20 Hz, 4 min). For phytoalexin analysis, homogenate was extracted with 300 μL 80% (v/v) aqueous methanol containing 0.08% (v/v) formate and 2.5 μL internal standard (200 μM 4-methoxyindole/4M-I [Sigma-Aldrich] in 100% methanol) per mg sample dry weight. Extracts were sonicated for 5 min and centrifuged at 16,000 × *g* for 2 min. The supernatant was filtered using a 0.45 μm polypropylene filter plate (GE Healthcare, Chicago, IL).

Samples were separated on an Ultimate 3000 HPLC (Dionex, Sunnyvale, CA) system, using a 3.5 μm, 3 × 150 mm Zorbax SB-Aq column (Agilent, Santa Clara, CA) with the gradient shown in Supplementary Table 7. A coupled DAD-3000RS diode array detector (Dionex), FLD-311 fluorescence detector (Dionex), and MSQPlus mass spectrometer (MS) (Dionex) collected UV absorption spectra in the range of 190–560 nm, a collected fluorescence data at 275/350 nm (ex/em), and collected electrospray ionization (ESI) mass spectra in positive and negative ion modes in the range of 100–1000 m/z, respectively. Total ICN, 4OH-ICN, and camalexin amounts were quantified using standard curves of standards prepared in *cyp79B2 cyp79B3* seedling extract and integrated areas in the UV chromatographs at 260 nm for 4M-I (retention time [RT] = 7.7 min); 340 nm for ICN (RT = 11.5 min); 280 nm for ICN degradation product ICA-ME (RT = 9.5 min), and co-eluting 4OH-ICN degradation products 4OH-ICA and 4OH-ICA-ME (RT = 10.1 min); and 320 nm for camalexin (RT = 12.1 min). For Fig. 1b, total camalexin amounts were quantified using integrated areas in the FLD chromatograph. For some experiments, 2.5 μL 200 μM indole butrytic acid (IBA; RT = 10.1 min) was added per mg sample dry weight instead of 4M-I. Relative amounts of ICN, 4OH-ICN, and amounts were quantified by dividing the peak areas at m/z 169 [M-H]^−^ (ICN), 174 [M-H]^−^ (ICA-ME), 176 [M-H]^−^ (4OH-ICA), 190 [M-H]^−^ (4OH-ICME), and 201 [M + H]^+^ (camalexin), by the peak area at m/z 202 [M-H]^−^ (IBA).

### Glucosinolate extraction and LC-DAD-FLD-MS

For glucosinolate extraction, a 96-well 0.45 μm polyvinylidene fluoride (PVDF) filter plate (EMD Millipore, Billerica, MA) was charged with 45 mg DEAE Sephadex A25 (GE Heathcare) and 300 μL of water per well, and equilibrated at room temp for 2 h. Prior to sample homogenization, the plate was centrifuged at 400 × *g* for 1 min to remove the water. The homogenate was extracted with 500 μL 70% (v/v) aqueous methanol at 67.5 °C for 10 min and centrifuged at 16,000 × *g* for 2 min. Added to the supernatant was 3 μL of IS (1.25 mM sinigrin (Sigma-Aldrich) in 80% (v/v) ethanol) per mg sample dry weight. Extract was applied to and incubated on the ion exchanger for 10 min. The sephadex resin was washed three times with 70% (v/v) methanol, three times with distilled deionized water (ddH_2_O), and two times with 20 mM sodium acetate (pH 5). Twenty microliters of 25 mg/mL aryl sulfatase (Type H1 from *Helix pomatia*, Sigma-Aldrich) was applied to and incubated on the sephadex resin at RT overnight. The plate was centrifuged at 400 × *g* for 1 min and desulfoglucosinolates were eluted from the sephadex resin by two 100 μL washes with 60% (v/v) methanol and two 100 μL washes with ddH_2_O. Eluate volume was reduced to 250–350 μL using an evaporator.

Samples were separated on an Ultimate 3000 HPLC system, using a 3.5 μm, 3 × 150 mm Zorbax SB-Aq column with the gradient shown in Supplementary Table 7. A coupled DAD-3000RS diode array detector, FLD-311 fluorescence detector, and MSQPlus mass spectrometer collected UV absorption spectra at 229 nm, fluorescence spectra at 275/350 nm (ex/em), and ESI mass spectra in positive/negative ion modes at 100–1000 m/z, respectively. Glucosinolates were quantified using integrated areas of desulfoglucosinolates in the UV chromatographs at 229 nm and published response factors^73^.

### ChIP and PCR

For ChIP experiments on *wrky33/DEX:WRKY33-flag* nuclear extracts, approximately two hundred and ten 9-day-old seedlings were inoculated with *Psta* to OD_600_ of 0.013 and co-treated with mock solution of dimethyl sulfoxide (M) or 20 μM dex (D) for 9 h. Total protein was extracted in 25 mL of Extraction buffer 1 (0.4 M sucrose, 10 mM Tris-Cl [pH 8], 10 mM MgCl_2_, 5 mM 2-mercaptoethanol, 0.1 mM AEBSF, Complete EDTA-free protease inhibitor cocktail [Roche]. After a 10 min fixing step with 1% (v/v) formaldehyde solution and a 5 min quenching step with 2 M glycine, seedlings were washed three times with deionized water, vacuum-dried, and snap-frozen with liquid nitrogen. Following frozen homogenization, the homogenate was filtered once through a 70 µm mesh (Carolina Biological) and a 0.45 µm filter (EMD Millipore). Filtered homogenate was then washed once in 500 µL of Extraction buffer 2 (0.25 M sucrose, 10 mM Tris-Cl [pH 8], 10 mM MgCl_2_, 1% [v/v] Triton X-100, 5 mM 2-mercaptoethanol, 0.1 mM AEBSF, Complete EDTA-free protease inhibitor cocktail) and resuspended in 300 µL of Extraction buffer 3 (1.7 M sucrose, 10 mM Tris-Cl [pH 8], 0.15% [v/v] Triton X-100, 2 mM MgCl_2_, 5 mM 2-mercaptoethanol, 0.1 mM AEBSF, Complete-Mini EDTA-free protease inhibitor cocktail) prior to sucrose centrifugation. Following nuclear extraction, samples were resuspended in 125 µL of Nuclei Lysis buffer (50 mM Tris-Cl [pH 8], 10 mM EDTA, 1% [v/v] SDS, 0.1 mM AEBSF, Complete-Mini EDTA-free protease inhibitor cocktail), and 250 µL of ChIP dilution buffer (1% [v/v] Triton X-100, 1.2 mM EDTA, 16.7 mM Tris-Cl [pH 8], 167 mM NaCl, Complete EDTA-free protease inhibitor cocktail), sonicated in a Covaris S2 sonicator (Covaris, Woburn, MA) using 10% duty, 7% intensity, 200 cycles per burst for a total time of 11 min, and centrifuged at 16,000 × *g* for 10 min at 4 °C to precipitate SDS. ChIP was performed using Anti-FLAG M2 Affinity Gel (Sigma-Aldrich). Beads were pre-treated with 0.1% (w/v) non-fat milk in 1× phosphate-buffered saline (PBS) and 0.5 mg/mL sheared salmon sperm DNA (Invitrogen). Following de-crosslinking, DNA samples were phenol-chloroform-extracted, diluted to the same OD_260_ concentration, and 1.5 μL was used in a 15 μL PCR reaction.

PCR analysis was performed on nuclear extracts prior to antibody incubation (input) and after ChIP. PCR conditions were as follows: 95 °C for 3 min; 40 cycles of 95 °C for 15 s, 53 °C for 15 s, and 72 °C for 1 min; 72 °C for 5 min. Densitometric determination of signal intensity in each ChIP and input sample was calculated using ImageJ. Fold enrichment was determined by calculating the ratio of PCR product intensities in ChIP D/M to Input D/M. In cases where amplicons were absent, an arbitrary value of 10 was assigned. For *EPL2*, qPCR analysis was additionally performed to confirm absence of amplicons in ChIP samples. RLU counts at the 25th cycle were used for quantification. Primer sequences are listed in Supplementary Data 2.

### SDS-PAGE and western blotting

Total protein was extracted from snap-frozen seedlings into 80 µL of extraction buffer (50 mM Tris-Cl [pH 7.5], 50 mM dithiothreitol, 4% [w/v] SDS, 10% [v/v] glycerol) using a 5 mm stainless steel bead and ball mill (20 Hz for 3 min). Samples were centrifuged briefly, incubated at 95 °C for 10 min, and centrifuged at 12,000 × *g* for 8 min to precipitate insoluble material.

Five (for WRKY33-flag) or 15 µL (for WRKY33-myc) of extract was loaded onto a 10% SDS-PAGE gel and the separated proteins were transferred to PVDF membrane (Millipore, Billerica, MA), stained with Ponceau S for labeling of total protein, and probed with either FLAG M2 (Sigma-Aldrich, cat# F1804) or c-Myc 9E10 (Santa Cruz Biotechnology, cat# sc-40) antibodies diluted 1:1000 or 1:750, respectively, in 1× PBS containing 5% (w/v) non-fat milk.

### Comparative genomics

All phylogenetic species trees were adapted from published data^74,75^. To generate phylogenetic maximum likelihood (ML) trees, sequences were aligned using MUSCLE in MEGA7^76^ and the JTT model (for CYP82C and LINE alignments) or Tamura-Nei model (for the *EPCOT3* alignment). Sequences for all genes with the description “non-LTR retrotransposon family (LINE)” (*n* = 263) were batch-downloaded from TAIR (https://arabidopsis.org). Of these, sequences containing intact reverse-transcriptase domains (PGPDG, LIPK, FRPISL, or FADD sequences; *n* = 126) were used for subsequent phylogenetic analysis (Supplementary Notes 1 and 2). Gaps were removed from the CYP82C alignment, leaving a total of 480 codons. Information on genomes used for synteny analysis is shown in Supplementary Table 8.

Selection estimates based on nonsynonymous-to-synonymous substitution ratios were calculated from the CYP82C ML tree. A Newick tree file was generated from this ML tree (Supplementary Fig. 4b and Supplementary Data 1) and for Branch site models, branches were pre-defined. CodeML analysis in PAML^77^ was then conducted with the following modified parameters: ncatG = 8, CodonFreq = 3. The M0 test was performed with model = 0 and NSsites = 0. The M1a-null test was performed with model = 0 and NSsites = 1. A more stringent null test (fixed omega) was performed for each Branch site model to be tested (model = 2 and NSsites = 2), where omega was fixed to 1. Branch site models were then tested with unfixed omega. Likelihood ratio tests were performed by comparing critical values and degrees of freedom between each unfixed Branch site test and either the M1a test or the corresponding fixed-omega test. Pre-defined branches with *P*-values < 0.05 for both tests were regarded as under positive selection (Supplementary Data 1).

### Bioinformatics

Epigenetics data were obtained from published work^55,56^. Percent identity matrices were constructed from Clustal Omega Multiple Sequence Alignments (https://www.ebi.ac.uk/Tools/msa/clustalo/). Promoter alignment plots were generated using mVISTA (http://genome.lbl.gov/vista/mvista/submit.shtml)^78^.

### Reporting Summary

Further information on research design is available in the Nature Research Reporting Summary linked to this article.

## Supplementary information

Supplementary Information

Peer Review

Reporting Summary

Description of Additional Supplementary Files

Supplementary Dataset 1

Supplementary Dataset 2

Source Data

## Data Availability

The authors declare that all data supporting the findings of this study are available within the paper and the Supplementary Information or are available from the corresponding authors upon request. A reporting summary for this article is available as a Supplementary Information file. The source data underlying Figs. 1b, 2b, 2c, 3a, 3c, 4a, 4c, 4d, 5d, and 5e, as well as Supplementary Figures 1b, 1c, 2a, 2c, 2d, 3a, 4b-f, 5a, 5b, 5d, 5e, 6a, 6c, 6e, 7d, and 7e are provided as a Source Data file.

## Code availability

Custom codes to visualize the CYP82C2 homology model in MacPyMOL and conduct statistics in R are available at https://github.com/brendenbarco.
