# Supplementary material for: Expansion of a core regulon by transposable elements promotes Arabidopsis chemical diversity and pathogen defense
Source: Nat Commun. 2019 Aug 1;10:3444. doi: 10.1038/s41467-019-11406-3 (PMC6671987; doi:10.1038/s41467-019-11406-3)
Supplement: Supplementary file 4 — Description of Additional Supplementary Files [file 41467_2019_11406_MOESM4_ESM.docx]

**Description of Additional Supplementary Files**

File Name: Supplementary Data 1
Description: PAML output for purifying and positive selection on CYP82C family

File Name: Supplementary Data 2
Description: Primer sequences used for qPCR, ChIP-PCR, and RT-PCR
