# Supplementary material for: Expansion of a core regulon by transposable elements promotes Arabidopsis chemical diversity and pathogen defense
Source: Nat Commun. 2019 Aug 1;10:3444. doi: 10.1038/s41467-019-11406-3 (PMC6671987; doi:10.1038/s41467-019-11406-3)
Supplement: Supplementary file 2 — Peer Review [file 41467_2019_11406_MOESM2_ESM.pdf]

Reviewers' comments:

Reviewer #1 (Remarks to the Author):

The manuscript by Barco and colleagues reports on the identification of a LINE element involved in the neo-functionalization of a gene onvolved in the synthesis of alcaloids in response to pathogens. The authors have previously contributed to show that 4OH-ICN is a new metabolite involved in the pathogen response, the product of the gene CYP82C2 (a cytochrome P450 enzyme) being responsible for its biosynthesis. Notably CYP82C2 is part of a cluster of 3 genes replicated in tandem and potentiallt neo-functionalized.
Here they show that the pathogen responsiveness of CYP82C2 is mediated by the WRKY33 transcription factor. Interestingly WRKY33 binding site in the CYP82C2 promoter is located within an unannotated fragment of a LINE element. The authors reconstruct the history of this LINE retrotransposition showing that its insertion upstream of CYC82C2 is specific to the Arabidopsis thaliana species. An Arabidopsis thaliana accession (Dijon) phenotyped for its low abundance of 4OH-ICN is further shown to be a 'natural' mutant for WRK33. Given that this mutation affects the fitness, the authors make the hypothesis that this ecotype could the product of a mutagenized Ler line.

The manuscript is very well written and the data support the conclusions. All experiments are clearly presented and executed. The authors have identified an example of the contribution of transposable elements in the regulation of gene expression that is important for the plant fitness. This example for its clarity and importance will likely be integrated in textbooks. I have no remark concerning the data but an important suggestion concerning the cited literature. As non TE specialists the authors might not be aware of the work of Lynch et al Nature Genetics 2011 or more recently Chuong et al (Feschotte group) Science 2016 showing that TEs can be involved in regulatory networks (placental development or interferon response in mammals). TE-mediated amplification of transcription binding sites as also been documented in Brassica (Henaff et al Plant J 2014). Nevertheless the present work represents a very well documented example of the impact of a TE on neo-functionalization of a tandem replicated gene.

Regarding the Dijon accession, Sup Table 7 suggests that some SNPs are "WT" in Dijon and not in Ler. How is this compatible with the hypothesis of Dijon being a EMS treated Ler? Do the 25000 SNPs between Ler and Dijon carry the features of EMS mutations?

Minor remarks:

Italicize mutant name in Fig 2
Statistics with capital letters not clearly understood in Fig 3
Scheme requires details in Fig4B (what are grey squares?)
Fig4D precise A thaliana
Supp Fig 2 and Supp Fig4F colors not visible in my PDF

Reviewer #2 (Remarks to the Author):

Brassicaceae including the model plant Arabidopsis thaliana possess a diverse Trp-derived metabolism that produces defense-related indolic compounds. The biosynthesis of several of these indolics, including the phytoalexin camalexin and 4-hydroxyindole-3-carbonitrile (4OH-ICN), is inducible upon pathogen attack and mainly produced in infected plant tissue. This study describes an evolutionary scenario of how Arabidopsis thaliana has acquired a pathogen-inducible 4OH-ICN biosynthetic pathway. A key step in this scenario is the integration of a transposable element (EPCOT3) into the A.

thaliana genome upstream of the 4OH-ICN biosynthetic gene CYP82C2. A sequence of EPCOT3 developed into a binding element for the transcription factor WRKY33, and this gave rise to the pathogen-inducible character of 4OH-ICN biosynthesis. The study is original and describes a novel evolutionary mechanism resulting in the inducible biosynthesis of a lineage-specific defense metabolite. Many of the conclusions are supported in my opinion by the experimental data provided. However, I have also concerns about the validity of some of the manuscript statements. I also think that the study requires improved experimentation and evaluation with respect to the following aspects:
- The study addresses pathogen-inducible plant responses. Many figures that present inducible metabolite or gene expression analyses thereby compare samples collected at the time of infection (0h) with samples collected at a certain time after infection (for example 12h or 24h samples; e.g. Fig. 4c, Suppl. Fig. 1b,c). Although the observed effects may well result from the pathogen-treatment, they might also result from other stimuli or parameters (such as Silwet- or day/night effects). The comparison of mock-treated samples with pathogen-treated samples at a given time-point would ascertain that the inducibility of genes or metabolites is due to the pathogen effect only.
- In the same context: Several data sets only present metabolite and gene expression data from pathogen-treated but not from uninfected (mock-treated) plants. (for example Fig.1b, Fig. 2a, Fig. 3a, Suppl. Fig. 2, Suppl. Fig. 3a). This also includes experiments with DEX-inducible promoter lines. Again, the mock-treatments are necessary in my opinion for a proper judgement of the pathogen-inducible responses under study. If metabolite values are below detection limit for control samples, this could be indicated directly in the figure.
- Statistical analyses: When values of more than two lines or treatments are compared (for example Fig. 2b,c), it is appropriate to use ANOVA rather than t-test
- Line 110/129: The statement that 4OH-ICN biosynthesis is specific to ETI (and does not occur in PTI) is not sufficiently supported in my opinion. Only one kind of PAMP (flg22) in one dose has been tested but no other PAMPs and/or no other doses. Also, only one compatible bacterial pathogen (Pst – which may specifically suppress 4OH-ICN biosynthesis) has been tested.
- Line 161: The statement „These results indicate that WRKY33 is required and sufficient to activate camalexin and 4OH-ICN biosynthesis‟ is also questionable. The results from Suppl. Fig. 2d indeed show that WRKY33 is required for a strong activation of camalexin/ICN/4OH-ICN biosynthesis. However, a smaller WRKY33-independent contribution also appears to exist, and the data does not support the conclusion that WRKY33 was sufficient for the responses (e.g. Pseudomonas infection will activate many other transcription factors that might be necessary for the response)
- Lines 164-201: ‟Intraspecific variation in WRKY33 affects 4OH-ICN synthesis and pathogen defense‟. This is a precarious statement because in the Di-G ecotype, variations other than in WRKY33 might cause to observed defense effects. A key experiment to solidify this statement would be to test whether expression of the Col-0 variant of WRKY33 in Di-G results in Col-0-like 4OH-ICN accumulation and disease resistance.
- Line 200: „Our results support a specific role of WRKY33 in pathogen defense as an activator of Trp-derived specialized metabolism‟. The presented data points to a role of WRKY33 in pathogen defense and Trp-derived specialized metabolism, but whether this role is specific for this metabolic pathway is far from clear
- Fig. 3a: FOX1 transcript levels are similar between WT and wrky33 plants, arguing against the conclusion that WRKY33 mediates expression of the 4OH-ICN biosynthetic gene FOX1.

Reviewer #3 (Remarks to the Author):

This manuscript described analysis of Arabidopsis WRKY33 in the regulation of three related defense metabolites with a special focus on 4OH-ICN. Although the title of the manuscript is on the role of a putative TE as an enhancer in the promoter of a 4OH-ICN biosynthetic gene, a majority of the study was actually focused on the role of WRKY33 in the biosynthesis of 4OH-ICN. The study has a substantial body of data that is interesting and can be potentially important. However, in my opinion

the study appeared to be quite preliminary with several sets of correlative data that do not establish causal relationships to the issues to be addressed. Some of the important conclusions, as a result, are not necessarily true or at least appear to be premature. Here are my main concerns:

1. Starting from line 131, the manuscript described the evidence that WRKY33 is required and sufficient to activate 4OH-ICN. The conclusion that WRKY33 is sufficient to activate biosynthesis of 4OH-ICN is clearly not correct because WRKY33 is induced under a variety of conditions including flg33 and Pst infection, under which 4OH-ICN biosynthesis is not activated. The described experiment was carried out in Psta-challenged wrky33 plants and it is unclear whether 4OH-ICN biosynthesis can also be activated in unchallenged plants after induction of WRKY33 gene. If the positive result is only found in Psta-challenged plants, it would indicate that WRKY33 plus additional factors generated during ETI are required.
2. Di-G has a mutation in the WRKY33 gene and the authors claimed that the mutation is responsible for the low levels of defense metabolites in the ecotype. In the part describing these results, it was indicated that they introduced the inducible WRKY33 construct into the wrky33 mutant and found restoration of WT levels of resistance (lines 198-201). However, nothing was mentioned on whether they also introduced the WRKY construct into Di-G accession and see whether it can also restore WT levels of the defense metabolites and resistance.
3. The last part of the manuscript on the CYP82C2 enhancer is very preliminary. Most the data are correlative from bioinformatic analysis, DNA-binding and epigenetic markers. There are important questions that can be addressed experimentally. First, they need to provide direct evidence that the lack of 4OH-ICN in other close species is due to absence of expression of their CYP82C2 genes. This can be tested by introduce the Arabidopsis CYP82C2 gene into these species. Second, they need data from promoter dissection that EPCOT3 element with W4 binding site is required for the expression of the CYP82C2 gene in Arabidopsis.

NCOMMS-18-25039 Expansion of a core regulon by transposable elements promotes Arabidopsis chemical diversity and pathogen defense by Barco B, Kim Y, Clay NK.

Reviewer #1:

The manuscript by Barco and colleagues reports on the identification of a LINE element involved in the neo-functionalization of a gene involved in the synthesis of alkaloids in response to pathogens. The authors have previously contributed to show that 4OH-ICN is a new metabolite involved in the pathogen response, the product of the gene CYP82C2 (a cytochrome P450 enzyme) being responsible for its biosynthesis. Notably CYP82C2 is part of a cluster of 3 genes replicated in tandem and potentially neo-functionalized.
Here they show that the pathogen responsiveness of CYP82C2 is mediated by the WRKY33 transcription factor. Interestingly WRKY33 binding site in the CYP82C2 promoter is located within an unannotated fragment of a LINE element. The authors reconstruct the history of this LINE retrotransposition showing that its insertion upstream of CYC82C2 is specific to the Arabidopsis thaliana species. An Arabidopsis thaliana accession (Dijon) phenotyped for its low abundance of 4OH-ICN is further shown to be a 'natural' mutant for WRKY33. Given that this mutation affects the fitness, the authors make the hypothesis that this ecotype could the product of a mutagenized Ler line.

The manuscript is very well written and the data support the conclusions. All experiments are clearly presented and executed. The authors have identified an example of the contribution of transposable elements in the regulation of gene expression that is important for the plant fitness. This example for its clarity and importance will likely be integrated in textbooks.

I have no remark concerning the data but an important suggestion concerning the cited literature. As non TE specialists the authors might not be aware of the work of Lynch *et al* Nature Genetics 2011 or more recently Chuong *et al* (Feschotte group) Science 2016 showing that TEs can be involved in regulatory networks (placental development or interferon response in mammals). TE-mediated amplification of transcription binding sites as also been documented in Brassica (Henaff *et al* Plant J 2014). Nevertheless the present work represents a very well documented example of the impact of a TE on neo-functionalization of a tandem replicated gene.
Thank you for these references. We have since incorporated them into our introductory paragraphs.

Regarding the Dijon accession, Sup Table 7 suggests that some SNPs are "WT" in Dijon and not in Ler. How is this compatible with the hypothesis of Dijon being a EMS treated Ler?
Do the 25000 SNPs between Ler and Dijon carry the features of EMS mutations?
We show in Supplementary Table 4 that Di-G does not appear to be enriched in features of EMS mutations (i.e. transition mutations) or X-ray mutations (i.e. indels) relative to closely related pseudogenomes.

Minor remarks:

Italicize mutant name in Fig 2
Statistics with capital letters not clearly understood in Fig 3
Scheme requires details in Fig4B (what are grey squares?)
Fig4D precise A thaliana
Supp Fig 2 and Supp Fig4F colors not visible in my PDF
Thank you for catching these mistakes. We have since corrected and/or clarified these issues. For Figure 2a, the unitalicized mutant name denotes a mutant protein, and in our field, all protein names are unitalicized.

Reviewer #2:

Brassicaceae including the model plant Arabidopsis thaliana possess a diverse Trp-derived metabolism that produces defense-related indolic compounds. The biosynthesis of several of these indolics, including the phytoalexin camalexin and 4-hydroxyindole-3-carbonitrile (4OH-ICN), is inducible upon pathogen attack and mainly produced in infected plant tissue. This study describes an evolutionary scenario of how Arabidopsis thaliana has acquired a pathogen-inducible 4OH-ICN biosynthetic pathway. A key step in this scenario is the integration of a transposable element (EPCOT3) into the A. thaliana genome upstream of the 4OH-ICN biosynthetic gene CYP82C2. A sequence of EPCOT3 developed into a binding element for the transcription factor WRKY33, and this gave rise to the pathogen-inducible character of 4OH-ICN biosynthesis. The study is original and describes a novel evolutionary mechanism resulting in the inducible biosynthesis of a lineage-specific defense metabolite. Many of the conclusions are supported in my opinion by the experimental data provided. However, I have also concerns about the validity of some of the manuscript statements. I also think that the study requires improved experimentation and evaluation with respect to the following aspects:
- The study addresses pathogen-inducible plant responses. Many figures that present inducible metabolite or gene expression analyses thereby compare samples collected at the time of infection (0h) with samples collected at a certain time after infection (for example 12h or 24h samples; e.g. Fig. 4c, Suppl. Fig. 1b,c). Although the observed effects may well result from the pathogen-treatment, they might also result from other stimuli or parameters (such as Silwet- or day/night effects). The comparison of mock-treated samples with pathogen-treated samples at a given time-point would ascertain that the inducibility of genes or metabolites is due to the pathogen effect only.
- In the same context: Several data sets only present metabolite and gene expression data from pathogen-treated but not from uninfected (mock-treated) plants. (for example Fig.1b, Fig. 2a, Fig. 3a, Suppl. Fig. 2, Suppl. Fig. 3a). This also includes experiments with DEX-inducible promoter lines. Again, the mock-treatments are necessary in my opinion for a proper judgement of the pathogen-inducible responses under study. If metabolite values are below detection limit for control samples, this could be indicated directly in the figure.
As a specificity control for ETI responses, we have now included the *rpm1* mutant, which cannot perceive the pathogen effector avrRpm1 but can perceive all other pathogen treatment effects. The *rpm1* control data (Supplementary Fig. 1b-c) shows that receptor-

mediated perception of avrRpm1 is required for *Psta*-induced camalexin and 4OH-ICN biosyntheses.
We have now added uninfected control data (Supplementary Fig. 2d, top row) to show that dexamethasone treatment alone yields no detectable levels of defense metabolites. Furthermore we have added a DMSO treated control with *Psta* to show that dex and *Psta* together are required to activate camalexin and 4OH-ICN biosynthesis in *wrky33/DEX:WRKY33-flag* lines (Supplementary Fig. 2d, bottom row).

- Statistical analyses: When values of more than two lines or treatments are compared (for example Fig. 2b,c), it is appropriate to use ANOVA rather than t-test
We are now applying one-factor ANOVAs coupled to Tukey's HSD test to experiments with more than two treatments or genotypes (Figs. 1b, 2b-c, 3a; Supplementary Figs. 2d, 3a, 4e), one exception being Fig. 4b-c, where we have instead limited the analyses to comparisons between stress-treated/untreated pairs for the t-test.

- Line 110/129: The statement that 4OH-ICN biosynthesis is specific to ETI (and does not occur in PTI) is not sufficiently supported in my opinion. Only one kind of PAMP (flg22) in one dose has been tested but no other PAMPs and/or no other doses. Also, only one compatible bacterial pathogen (Pst – which may specifically suppress 4OH-ICN biosynthesis) has been tested.
In addition to flg22, we have now added elf26 peptide and chitosan treatments at concentrations previously reported to saturate receptor-mediated immune responses (Felix *et al*., 1999 Plant Journal 18:265; Zipfel *et al*., 2006 Cell 125:749; Povero *et al*., 2011). We observed that flg22 and elf26 do not significantly induce the accumulation of camalexin or 4OH-ICN in seedlings (Fig. 1b). However, we did observe that 300 µg/mL chitosan induced strong camalexin and ICN levels, as well as detectable 4OH-ICN. High chitosan concentrations have been shown to induce hypersensitive response (HR)-like cell death in Arabidopsis (Cabrera *et al*., 2006 Physiol. Plant.), a phenomenon commonly observed for ETI (Jones and Dangl, 2006 Nature), thus we conclude that classical PTI responses may not be exclusively triggered in response to this treatment. As per your recommendation, we also added data from treatments with additional compatible and incompatible *P. syringae* strains as well as ETI-inducing pathogen effectors for a total of seven different *P. syringae* lines (2 compatible and 5 incompatible) and three pathogen effectors (avrRpm1, avrRps4, and avrRpt2) to show that ETI-like responses trigger 4OH-ICN biosynthesis.

- Line 161: The statement "These results indicate that WRKY33 is required and sufficient to activate camalexin and 4OH-ICN biosynthesis" is also questionable. The results from Suppl. Fig. 2d indeed show that WRKY33 is required for a strong activation of camalexin/ICN/4OH-ICN biosynthesis. However, a smaller WRKY33-independent contribution also appears to exist, and the data does not support the conclusion that WRKY33 was sufficient for the responses (e.g. Pseudomonas infection will activate many other transcription factors that might be necessary for the response)
We have added the *rpm1* control data to show that a minor WRKY33-independent contribution also exists (Supplemental Fig. 1b-c). We changed the text to state that

induced expression of *WRKY33-flag* restored camalexin and 4OH-ICN biosynthesis in *Psta*-challenged *wrky33* plants to WT levels or greater and that *WRKY33* is thus required to activate camalexin and 4OH-ICN biosynthesis in response to *Psta.*

- Lines 164-201: "Intraspecific variation in WRKY33 affects 4OH-ICN synthesis and pathogen defense". This is a precarious statement because in the Di-G ecotype, variations other than in WRKY33 might cause the observed defense effects. A key experiment to solidify this statement would be to test whether expression of the Col-0 variant of WRKY33 in Di-G results in Col-0-like 4OH-ICN accumulation and disease resistance.
We have now added text to the Results section and data to show that WRKY33 with a C-terminal 6x-myc tag (WRKY33-myc) restored *CYP71A13/CYP71B15/CYP71A12/FOX1* expression, camalexin biosynthesis, and *Pst* resistance in Col-0 *wrky33* to WT levels (Fig. 2c, Supplementary Fig. 4a-d,f). Importantly, in Di-G it similarly restored camalexin biosynthesis and *Pst* resistance to L*er*-1 (WT) levels (Fig. 2c, Supplementary Fig. 4e). Although WRKY33-myc activated biosynthesis of ICN (precursor to 4OH-ICN) in Col-0 *wrky33* and Di-G to greater than WT levels, it did not activate *CYP82C2* expression and 4OH-ICN biosynthesis in Col-0 *wrky33* and Di-G to WT levels (Supplementary Fig. 4d-f). Because the C-terminal domain of WRKY33 is absolutely required for stress responses (Zhou *et al*., 2015 J Exp Bot), we hypothesized that the large 6x-myc tag at the C-terminus interfered with the activation of the *CYP82C2* gene. We subsequently generated the construct featuring WRKY33 with 1x-flag tag (WRKY33-flag), which we found to activate *CYP82C2* expression and 4OH-ICN biosynthesis in Col-0 *wrky33* to WT levels (Supplementary Fig. 2d, Fig. 3a). Although we did not introduce the WRKY33-flag construct into Di-G, our results from the WRKY33-myc construct in Di-G and Col-0 *wrky33* demonstrate that the *wrky33* mutation in Di-G is indeed responsible for the camalexin and pathogen susceptibility phenotypes previously reported for this accession.

- Line 200: "Our results support a specific role of WRKY33 in pathogen defense as an activator of Trp-derived specialized metabolism". The presented data points to a role of WRKY33 in pathogen defense and Trp-derived specialized metabolism, but whether this role is specific for this metabolic pathway is far from clear
We have corrected this section to indicate that activation of Trp-derived metabolism is not the exclusive/specific role of WRKY33.

- Fig. 3a: FOX1 transcript levels are similar between WT and wrky33 plants, arguing against the conclusion that WRKY33 mediates expression of the 4OH-ICN biosynthetic gene FOX1.
We repeated this experiment using more biological replicates. The inclusion of these revised data in Fig. 3a as well as additional datasets from WRKY33-myc experiments (Supplementary Fig. 4f) indicates that *FOX1* expression is slightly but significantly reduced in the *wrky33* mutant, consistent with WRKY33-dependent regulation.

Reviewer #3:

This manuscript described analysis of Arabidopsis WRKY33 in the regulation of three related defense metabolites with a special focus on 4OH-ICN. Although the title of the manuscript is on the role of a putative TE as an enhancer in the promoter of a 4OH-ICN biosynthetic gene, a majority of the study was actually focused on the role of WRKY33 in the biosynthesis of 4OH-ICN. The study has a substantial body of data that is interesting and can be potentially important. However, in my opinion the study appeared to be quite preliminary with several sets of correlative data that do not establish causal relationships to the issues to be addressed. Some of the important conclusions, as a result, are not necessarily true or at least appear to be premature. Here are my main concerns:

1. Starting from line 131, the manuscript described the evidence that WRKY33 is required and sufficient to activate 4OH-ICN. The conclusion that WRKY33 is sufficient to activate biosynthesis of 4OH-ICN is clearly not correct because WRKY33 is induced under a variety of conditions including flg33 and Pst infection, under which 4OH-ICN biosynthesis is not activated. The described experiment was carried out in Psta-challenged wrky33 plants and it is unclear whether 4OH-ICN biosynthesis can also be activated in unchallenged plants after induction of WRKY33 gene. If the positive result is only found in Psta-challenged plants, it would indicate that WRKY33 plus additional factors generated during ETI are required.
As a specificity control for ETI responses, we have now included the *rpm1* mutant, which cannot perceive the pathogen effector avrRpm1 but can perceive all other effects from pathogen treatment. The *rpm1* control data (Supplementary Fig. 1b-c) shows that receptor-mediated avrRpm1 perception is required for *Psta*-induced camalexin and 4OH-ICN biosyntheses.
We have also now added uninfected control data (Supplementary Fig. 2d, top row) to show that dexamethasone treatment alone yields no detectable levels of metabolites. Furthermore we have added a DMSO treated control with *Psta* to show that dex and *Psta* together are required to activate camalexin and 4OH-ICN biosynthesis in *wrky33/DEX:WRKY33-flag* lines (Supplementary Fig. 2d, bottom row).

2. Di-G has a mutation in the WRKY33 gene and the authors claimed that the mutation is responsible for the low levels of defense metabolites in the ecotype. In the part describing these results, it was indicated that they introduced the inducible WRKY33 construct into the wrky33 mutant and found restoration of WT levels of resistance (lines 198-201). However, nothing was mentioned on whether they also introduced the WRKY construct into Di-G accession and see whether it can also restore WT levels of the defense metabolites and resistance.
We have now added text to the Results section and data to show that WRKY33 with a C-terminal 6x-myc tag (WRKY33-myc) restored *CYP71A13/CYP71B15/CYP71A12/FOX1* expression, camalexin biosynthesis, and *Pst* resistance in Col-0 *wrky33* to WT levels (Fig. 2c, Supplementary Fig. 4a-d,f). Importantly, in Di-G it similarly restored camalexin biosynthesis and *Pst* resistance to L*er*-1 (WT) levels (Fig. 2c, Supplementary Fig. 4e). Although WRKY33-myc activated biosynthesis of ICN (precursor to 4OH-ICN) in Col-0 *wrky33* and Di-G to greater than WT levels, it did not activate *CYP82C2* expression and 4OH-ICN biosynthesis in Col-0 *wrky33* and Di-G to WT levels (Supplementary Fig. 4d-f). Because the C-terminal domain of WRKY33 is absolutely required for stress responses

(Zhou *et al*., 2015 J Exp Bot), we hypothesized that the large 6x-myc tag at the C-terminus interfered with activation of *CYP82C2*. We subsequently generated the construct featuring WRKY33 with a 1x-flag tag (WRKY33-flag), which we found to activate *CYP82C2* expression and 4OH-ICN biosynthesis in Col-0 *wrky33* to WT levels (Supplementary Fig. 2d, Fig. 3a). Although we did not introduce the WRKY33-flag construct into Di-G, our results from the WRKY33-myc construct in Di-G and Col-0 *wrky33* demonstrate that the *wrky33* mutation in Di-G is indeed responsible for the camalexin and pathogen susceptibility phenotypes previously reported for this accession.

3a. The last part of the manuscript on the CYP82C2 enhancer is very preliminary. Most the data are correlative from bioinformatic analysis, DNA-binding and epigenetic markers. There are important questions that can be addressed experimentally. First, they need to provide direct evidence that the lack of 4OH-ICN in other close species is due to absence of expression of their CYP82C2 genes. This can be tested by introduce the Arabidopsis CYP82C2 gene into these species.
Synteny and phylogenetic analyses in Fig. 4b and Supplementary Fig. 6b show that *CYP82C2* genes are only present in *Arabidopsis* species. qPCR analyses in Fig. 4c show that *A. lyrata CYP82C2* and *CYP82C4* genes (i.e. all *A. lyrata CYP82C* genes) are not co-regulated with *A. lyrata CYP79B2/CYP71A12/FOX1* genes in pathogen defense. Our qPCR analysis in Fig. 4d shows that *A. lyrata CYP82C2* is also not co-regulated with sideretin biosynthetic genes *A. lyrata CYP82C4* and *F6'H1* in iron stress. Together, these results indicate that *A. lyrata CYP82C2* is in neither the ICN biosynthetic regulon in pathogen defense nor the sideretin biosynthetic regulon in iron stress. We confirmed this expression data with metabolic profiling data in Fig. 4a and Supplementary Fig. 6a to show that *A. lyrata* synthesizes ICN but not 4OH-ICN. Furthermore, we previously reconstituted the ICN biosynthetic pathway (minus *A. thaliana CYP82C2*) and the 4OH-ICN biosynthetic pathway (plus *CYP82C2*) in tobacco, a distantly related plant species that does not possess any clear *CYP82C* genes and does not synthesize ICN and 4OH-ICN (Rajniak *et al*., 2015). We showed that *CYP82C2* expression is required for 4OH-ICN biosynthesis in an engineered ICN-synthesizing plant species (Rajniak *et al*., 2015), providing direct evidence that the lack of *CYP82C2* expression is responsible for the lack of 4OH-ICN. Taken together, we believe that introducing the *A. thaliana CYP82C2* gene into the closely related species *A. lyrata,* which already has a *CYP82C2* gene, will not provide the requested evidence connecting gene expression to metabolite production.

3b. Second, they need data from promoter dissection that EPCOT3 element with W4 binding site is required for the expression of the CYP82C2 gene in Arabidopsis.
We evaluated *EPCOT3* function based on the four criteria for transposon-derived enhancers and promoters as outlined in de Souza *et al*. (2013), namely that the TE-derived sequence 1) is bound by a TF, 2) can increase transcription of a nearby gene, 3) has physiological function, and 4) has fitness effects. In addition, we evaluated *EPCOT3* based on classical criteria for enhancers, such as correlative histone modifications and by way of experimental analysis of *wrky33/DEX:WRKY33-flag* lines. In the latter case, we provide evidence linking induction of *CYP82C2* expression with WRKY33-*EPCOT3*

interaction (Fig. 3a,c). Our ChIP-PCR and epigenetic analyses in Fig. 5b and Fig. 5d altogether show that *EPCOT3* functions as an enhancer and not as a basal promoter. Furthermore, *EPL1* and *EPL2* (which have up to 85.4% sequence identity with *EPCOT3* and which share one or both of *EPCOT3*'s WRKY33-binding elements) do not bind as well as or at all with WRKY33, indicating that chromatin remodeling plays an important role in the development of the *EPCOT3* enhancer.

Loss-of-function studies such as enhancer dissections are rare and typically performed in the native chromatin environment, requiring homologous recombination-based gene targeting and precluding ectopic heterologous assays (e.g. protoplast transformation of reporter constructs). Gene targeting in higher plants, including *Arabidopsis thaliana*, remains challenging because of the extremely low efficiency of homologous recombination (Fauser *et al*., 2012 PNAS, Li *et al*., 2013 Nat. Biotech). Our current attempts to utilize the more efficient non-homologous end joining process for generation of targeted knock-outs for other genes of interest (LeBlanc *et al*., 2017 Plant Journal) have been challenging and time-consuming (> 1 year), and protocol papers on efficient gene targeting in plants are still being published in high-impact journals (e.g., Miki *et al*., 2018 Nat Commun). Finally, CRISPR-Cas9, which is the most developed genome editing technology in plants, is evolutionarily related to at least 5 classes of transposons (Koonin & Makarova, 2017 Genome Biol Evol) and has never been used to target an expanding transposon family of highly related members with the potential to retroduplicate under stress. For these reasons, we believe that the proposed line of experimentation is unfeasible for this study. Please note that only a handful of loss-of-function/enhancer dissection studies have been published (e.g., Bender *et al*., 2000 Mol Cell; Johnson *et al*., 2012 J Clin Invest; Bauer *et al*., 2013 Science; Gröschel *et al*., 2014 Cell; Mansour *et al*., 2014 Science; Canver *et al*., 2015 Nature), and to the best of our knowledge, none involve gene targeting in plants or transposon-derived enhancers/promoters in any organism.

Additional changes reported:

-Data for proCYP82C2 W2 and W1 (Figure 3c, Supplementary Figure 5b) was mistakenly switched, this has now been corrected. Additionally, new input images for proCYP82C2 W2 are now shown.

-In Figure 6, we have added question marks to the hypothesized iron stress role of the *A. lyrata CYP82C4* gene, as we have not directly tested iron stress responses in this species.

Reviewers' comments:

Reviewer #1 (Remarks to the Author):

The authors have addressed my comments. However when citing the references on TE-rewiring of gene networks in mammals they wrote that "the physiological significance of this rewiring, if any, is still unknown" (line 48). While this is true for plants, in mammals the functionnal significance has been clearly demonstrated, at least in the cited Chuong et al paper. Please clarify this point in the text.

Reviewer #2 (Remarks to the Author):

The authors have improved the manuscript significantly and overall addressed my previous comments in an adequate manner. I have no further comments.

Reviewer #3 (Remarks to the Author):

I raised a number of concerns on the original version of the manuscript. In this version of revision, the authors have provided new data with regards to my concern on whether WRKY33 is not only required but also sufficient for activated biosynthesis of 4OH-ICN. These data proved that the authors' original conclusion was in fact incorrect. The authors, however, have not made efforts to provide experimental data to address my other concerns, particularly the one concerning the lack of direct evidence that the lack of 4OH-ICN in other close species is due to absence of WRKY33-dependent/EPCOT3-mediated expression of their CYP82C2 genes.

As I indicated in my last review, a majority of this study was focused on WRKY33 in the biosynthesis of 4OH-ICN. This part is just confirmatory to the study published a few years ago by Shuqun Zhang's group on the role WRKY33 in the biosynthesis of camalexin. They have shown that WRKY33 is required for activated expression of a number of genes including CYP71A13 in the chorismite-to-IAN pathway, which is required for biosynthesis of both camalexin and ICN. The more interesting part of the manuscript is about the role of a putative TE inserted into the upstream region of the CYP82C2 gene. In their response letter, the authors argued that they have provided evidence that expression of CYP82C2 is required for 4OH-ICN biosynthesis. CYP82C2 is required for biosynthesis of 4OH-ICN and therefore, its expression, of course, is necessary for the production of the defense metabolite. The question they need to address is whether the difference in expression of CYP82C2 between A. thaliana and its close species A. lyrate is caused by the TE. I was not asking them to introduce the coding sequence of the CYP82C2 gene from A. thaliana into A. lyrate, which would be meaningless since the later species already has the gene. Instead, I was suggesting that they should isolate the genomic sequence of the CYP82C2 gene including the EPCOT3 elements from A. thaliana and introduce it into A. lyrate to see whether the introduced CYP82C2 gene from A. thaliana is expressed and 4OH-ICN biosynthesis is activated in the A. lyrate background.

NCOMMS-18-25039A Expansion of a core regulon by transposable elements promotes Arabidopsis chemical diversity and pathogen defense by Barco B, Kim Y, Clay NK.

Reviewer #1:

The authors have addressed my comments. However when citing the references on TE-rewiring of gene networks in mammals they wrote that "the physiological significance of this rewiring, if any, is still unknown" (line 48). While this is true for plants, in mammals the functional significance has been clearly demonstrated, at least in the cited Chuong et al paper. Please clarify this point in the text.

Thank you for catching this error in our wording. We have since moderated our statement to claim that general understandings of the physiological significance of TE-mediated exaptations are greatly limited, rather than unknown.

Reviewer #3:

I raised a number of concerns on the original version of the manuscript. In this version of revision, the authors have provided new data with regards to my concern on whether WRKY33 is not only required but also sufficient for activated biosynthesis of 4OH-ICN. These data proved that the authors' original conclusion was in fact incorrect. The authors, however, have not made efforts to provide experimental data to address my other concerns, particularly the one concerning the lack of direct evidence that the lack of 4OH-ICN in other close species is due to absence of WRKY33-dependent/EPCOT3-mediated expression of their CYP82C2 genes.

As I indicated in my last review, a majority of this study was focused on WRKY33 in the biosynthesis of 4OH-ICN. This part is just confirmatory to the study published a few years ago by Shuqun Zhang's group on the role of WRKY33 in the biosynthesis of camalexin. They have shown that WRKY33 is required for activated expression of a number of genes including CYP71A13 in the chorismate-to-IAN pathway, which is required for biosynthesis of both camalexin and ICN. The more interesting part of the manuscript is about the role of a putative TE inserted into the upstream region of the CYP82C2 gene. In their response letter, the authors argued that they have provided evidence that expression of CYP82C2 is required for 4OH-ICN biosynthesis. CYP82C2 is required for biosynthesis of 4OH-ICN and therefore, its expression, of course, is necessary for the production of the defense metabolite. The question they need to address is whether the difference in expression of CYP82C2 between A. thaliana and its close species A. lyrata is caused by the TE. I was not asking them to introduce the coding sequence of the CYP82C2 gene from A. thaliana into A. lyrata, which would be meaningless since the later species already has the gene. Instead, I was suggesting that they should isolate the genomic sequence of the CYP82C2 gene including the EPCOT3 elements from A. thaliana and introduce it into A. lyrata to see whether the introduced CYP82C2 gene from A. thaliana is expressed and 4OH-ICN biosynthesis is activated in the A. lyrata background.

To address the question of whether the *CYP82C2* locus of *A. thaliana* and not of *A. lyrata* is sufficient to activate gene expression, we have carried out transactivation experiments in tobacco leaves as well as in protoplasts of the *A. thaliana cyp82C2* mutant. We chose these systems because we viewed creation of transgenics in *A. lyrata* as relatively time-prohibitive due to difficulties related to self-incompatibility and plant propagation. For these experiments, we isolated *CYP82C2* genomic sequences from both *A. thaliana* and *A. lyrata* (ranging from 3000 bases upstream of start codon to stop codon). In the case of the *A. thaliana* sequence, this meant that the *EPCOT3* elements were also isolated. Introduction of these sequences into tobacco and protoplast alongside a dexamethasone-inducible copy of *AtWRKY33-flag* allowed us to test the effect of *EPCOT3* on *CYP82C2* gene expression. In the presence of dexamethasone and flg22, we observed WRKY33-mediated gene activation in tissues transfected with the *CYP82C2* locus of *A. thaliana*, but not of *A. lyrata* (Fig. 5d, Supplementary Fig. 7d), demonstrating that the *A. lyrata CYP82C2* locus, which does not include *EPCOT3*, is not responsive to WRKY33 activation.

Additional changes reported (see red for all changes):
-Minor wording/reference changes (Lines 72-74, 129).
-Additions to vector construction/transformation and RT-PCR analysis in Methods, primers to Supp. Table 6.

REVIEWERS' COMMENTS:

Reviewer #3 (Remarks to the Author):

The authors have addressed a critical concern I had with additional experiments using protoplast transfection.
